# Expansion of a food composition database for the food frequency questionnaire in the Korean Genome and Epidemiology Study (KoGES): a comprehensive database of dietary antioxidants and total antioxidant capacity

**DOI:** 10.4178/epih.e2024050

**Published:** 2024-05-10

**Authors:** Jiseon Lee, Ji-Sook Kong, Hye Won Woo, Mi Kyung Kim

**Affiliations:** 1 Department of Preventive Medicine, Hanyang University College of Medicine, Seoul, Korea; 2 Institute for Health and Society, Hanyang University, Seoul, Korea

**Keywords:** Antioxidants, Flavonoids, Vitamins, Database

## Abstract

**OBJECTIVES:**

This study constructed a comprehensive database of dietary antioxidants and total antioxidant capacity (TAC) to facilitate the estimation of daily antioxidant intake using a food frequency questionnaire (FFQ). This database was applied to 3 general population-based cohorts (n=195,961) within the Korean Genome and Epidemiology Study (KoGES).

**METHODS:**

To establish a database of 412 foods derived from recipes of a 106-item FFQ, we followed a pre-established standardized protocol. This included the selection of source databases, matching of foods, substitution of unmatched items with identical foods and input of values, and assessment of coverage. For each food, the TAC was estimated by summing the individual antioxidant capacities, calculated by multiplying the amount of each antioxidant by its vitamin C equivalent antioxidant capacity.

**RESULTS:**

We identified 48 antioxidants across 5 classes: retinol, carotenoids, vitamins C and E, and flavonoids, with flavonoids divided into 7 subclasses. TAC values were then established. Coverage exceeded 90.0% for retinol, carotenoids, vitamin C, and vitamin E, while coverage for flavonoids was 60.9%. The daily intakes of 4 antioxidant classes—all but vitamin E—were higher in women than in men. The Ansan-Ansung cohort exhibited the highest levels of dietary TAC, vitamin E, and flavonoids, while the Health Examinees Study cohort displayed the highest values for retinol, carotenoids, and vitamin C.

**CONCLUSIONS:**

We customized a comprehensive antioxidant database for the KoGES FFQ, achieving relatively high coverage. This expansion could support research investigating the impact of dietary antioxidants on the development of chronic diseases targeted by the KoGES.

## INTRODUCTION

Globally, epidemiological studies that investigate the role of diet in the development of chronic diseases typically employ a structured food frequency questionnaire (FFQ) to collect dietary information. Consequently, it is essential to develop databases tailored to the FFQ in use, designed to facilitate the calculation of both nutrient and non-nutrient intake levels pertinent to specific research objectives [1]. The capacity to concurrently quantify a wide array of food constituents associated with chronic diseases and their related traits, rather than focusing on a limited set of constituents, is critical. Fortunately, the limited number of foods in an FFQ, in contrast to open-ended dietary assessment methods such as 24-hour recall, enables the practical creation of a comprehensive database that includes the relevant food constituents. For example, a Harvard University database for their FFQ contains information on 227 constituents [2].

The Korean Genome and Epidemiology Study (KoGES), a representative large-scale prospective cohort study in Korea, encompasses 3 population cohorts and 3 gene-environment model studies. It was established with the goal of identifying modifiable lifestyle factors (such as diet) and genetic characteristics that contribute to common chronic diseases, in an effort to reduce the disease burden [3]. Dietary data from 3 population-based cohorts were collected using an FFQ that included 106 food items. However, the nutrient-related information derived from this study is limited to only 23 nutrients. Despite this limitation, a few studies have augmented the dataset by integrating external databases that provide additional information on fatty acids [4], glycemic index and glycemic load [5], copper [6], isoflavones [7,8], and flavonoids [9].

Oxidative stress results from an imbalance between reactive oxygen species and antioxidants. This phenomenon is implicated as a causative or associated risk factor for a variety of diseases in humans, including cardiovascular diseases (CVDs), cancer, and neurodegenerative disorders [10]. The human body employs multiple mechanisms to counter oxidative stress through naturally produced (endogenous) antioxidants, as well as those obtained from dietary sources (exogenous). Exogenous antioxidants have recently garnered considerable attention due to their potential to prevent or mitigate oxidative stress. The most recognized antioxidants include vitamin A (retinol), vitamin C (ascorbic acid), vitamin E (tocopherol), carotenoids (β-carotene), and polyphenols, such as flavonoids [11]. However, evidence regarding the prospective relationship between these antioxidants and diseases like chronic obstructive pulmonary disease [12], CVDs, and cancers [13] remains limited or inconsistent. This variability may stem in part from differences in antioxidant content among foods, as well as the differing antioxidant capacities of these compounds [14]. Consequently, the concept of dietary total antioxidant capacity (dTAC) has been introduced. dTAC represents a composite measure of the antioxidant capacity from all consumed foods [15], considering the synergistic interactions among antioxidants and their variable effectiveness in combating oxidation.

The KoGES database initially included only 5 components: vitamin A, retinol, carotene, and vitamins C and E. Considering the common complex diseases targeted by the KoGES, such as CVDs, cancer, type 2 diabetes, hypertension, obesity, metabolic syndrome (MetS), and osteoporosis, it is essential to prioritize the inclusion of food antioxidants in the database. The present study aimed to expand the KoGES FFQ database to incorporate 48 antioxidants. These include 5 classes of antioxidants (retinol, carotenoids, vitamins C and E, and flavonoids) and 7 subclasses of flavonoids (flavonols, flavones, flavanones, flavan-3-ols, anthocyanins, isoflavones, and proanthocyanidins); additionally, total antioxidant capacity (TAC) was calculated. We then estimated the daily intake of these antioxidants across 3 general population cohorts within the KoGES.

## MATERIALS AND METHODS

### Development of an antioxidant and total antioxidant capacity database for the Korean Genome and Epidemiology Study food frequency questionnaire

#### List of foods for the database

The dietary assessment method used in the KoGES is a semiquantitative FFQ, developed and validated by the Korea National Institute of Health [16]. This FFQ consists of 106 food items (Supplementary Material 1), with 9 frequency categories that range from “never or rarely” to “3 times per day”. Additionally, each food item is associated with 3 or 4 specified serving sizes. For seasonal foods, information on the duration of consumption was collected using 4 categories: 3 months, 6 months, 9 months, and 12 months. Initially, 475 foods were considered based on their recipes. However, after excluding duplicates, we included 412 unique foods in our analysis (Supplementary Material 2).

#### Establishing an antioxidant and TAC database: procedures for selecting and inputting antioxidant values

To construct this antioxidant and TAC database, hereafter referred to as the “antioxidant database,” it was crucial to employ standardized procedures. The establishment of the database, encompassing the selection of the source databases, adhered to a pre-established standardized protocol. This protocol was designed in line with the Food and Agriculture Organization/International Network of Food Data Systems (FAO/INFOODS) guidelines for food matching [17] (Supplementary Material 3).

For our source databases, we selected only food composition databases provided by government entities or authorized institutions. Our preference was the Korean database; however, in instances when this was not possible, we chose replacements based on the variety of antioxidants and available food items. For flavonoids, which accounted for 37 of the 48 antioxidants studied, we were unable to use the Korean database [18]. This was because most epidemiologic studies estimate flavonoid intake using the physiologically active aglycone form [19], but the Korean database lacked aglycone equivalents for some glucosides. We then considered a Japanese database, given Japan’s geographic proximity to Korea and the similarities in climate and food culture. However, this database did not include flavonoid data. Instead, we utilized 4 databases: the United States Department of Agriculture (USDA) Database for the Flavonoid Content of Selected Foods, release 3.3 [20]; the USDA Database for the Isoflavone Content of Selected Foods, release 2.1 [21]; and the USDA Database for the Proanthocyanidin Content of Selected Foods, release 2.1 [22]; followed by the Phenol-Explorer 3.6 database from the Institut National de la Recherche Agronomique [23]. The USDA databases provided flavonoid content in the aglycone form [20-22], while the PhenolExplorer database presented glycoside, ester, or aglycone forms [23]. When using data from the Phenol-Explorer database, we converted glycosides to aglycones using analytical procedures and molecular weights from an open chemistry database at the US National Institutes of Health [24]. For vitamins and carotenoids, we used 3 databases in the following order: the Food and Nutrient Database of the Korea Ministry of Food and Drug Safety [25]; the USDA National Nutrient Database for Standard Reference, release 28 [26]; and the Standard Tables of Food Composition from the Japanese Ministry of Education, Culture, Sports, Science and Technology, 7th revised edition [27]. Given the priority status of the USDA database for flavonoids, we also selected it over the Japanese database for information on vitamins and carotenoids.

The 412 foods from the FFQ were matched with corresponding items in the databases using the following process. First, when foods had identical names (either in Korean or English, including general names and cultivars) across databases, we chose antioxidant values for the same form (raw, dried, or boiled). If the variety of a food could not be determined, we selected an option based on the region of cultivation, domestic production of each variety, or variety most frequently consumed. For instance, for raw potatoes, we determined the domestic import quantities and consequently selected “Potato, *Sumi*, Raw” over “Potato, *Daeji*, Raw” from the Food and Nutrient Database of the Korea Ministry of Food and Drug Safety [25]. When data were scarce or it was difficult to ascertain the superiority of one variety over another, we used the average antioxidant values for all varieties of the food item. Second, for foods with the same general or scientific name but in different forms, we calculated antioxidant values using conversion factors that accounted for water content differences. This approach was primarily used for dried, boiled, and blanched forms. Third, when no foods with identical names were available, we assigned antioxidant values from similar foods based on criteria such as belonging to a similar species within the same genus or family, originating from the same plant part (leaf, stem, or root), or sharing the same color. Fourth, for prepared foods, we used recipes typically utilized by Koreans [28] and determined antioxidant values by summing the values of the individual ingredients in the recipe. Finally, based on a review of the literature, when specific antioxidants were known to be exclusive to certain food groups, we assigned a logical zero value to other groups not expected to contain these antioxidants; this primarily impacted animal-based food groups such as beef, pork, poultry, dairy products, meats, fish, processed meats, oils, and fats [29]. Consequently, we attributed zero values to the carotenoid and flavonoid contents of these animal-based food groups (Supplementary Material 4). Theaflavins, found only in tea products like green and black tea, led us to assign a zero value to all foods other than beverages and drinks [30]. Two trained nutritionists were responsible for selecting the appropriate foods to match the 412 items in the database. In instances of disagreement, the input of a third nutritionist was sought.

The study included 48 antioxidants: retinol, α-carotene, β-carotene, lycopene, β-cryptoxanthin, lutein and zeaxanthin, vitamin C, α-tocopherol, β-tocopherol, γ-tocopherol, δ-tocopherol, quercetin, kaempferol, myricetin, isorhamnetin, luteolin, apigenin, hesperetin, naringenin, eriodictyol, catechin, epicatechin, epigallocatechin, epicatechin 3-gallate, epigallocatechin 3-gallate, gallocatechin, theaflavin, thearubigin, theaflavin 3-gallate, theaflavin 3´- gallate, theaflavin 3,3´-digallate, cyanidin, delphinidin, malvidin, pelargonidin, peonidin, petunidin, isoflavone, daidzein, genistein, glycitein, biochanin, formononetin, dimers, trimers, 4-6 monomers, 7-10 monomers, and polymers. The analysis encompassed 5 antioxidant classes—retinol, vitamin C, vitamin E, carotenoids, and total flavonoids—as well as 7 subclasses of flavonoids: flavonols, flavones, flavanones, flavan-3-ols, anthocyanins, isoflavones, and proanthocyanidins (Table 1).

#### Estimation of TAC values for the 412 foods

To estimate the TAC value for each food in the antioxidant database, we utilized the vitamin C equivalent antioxidant capacity (VCEAC). This was measured using the 2,2´-azino-bis-3-ethylbenzthiazoline-6-sulfonic acid assay [31], and we adhered to the previously suggested theoretical method for estimating TAC [15]. In brief, the TAC of a food item was calculated by summing the antioxidant capacities, which were determined by multiplying the content of each antioxidant by its corresponding VCEAC value: $\Sigma \;\left[\left(\mathrm{antioxidant}\;\mathrm{content}\;\frac{mg}{100\;g}\;*\;\mathrm{antioxidant}\;\;\mathrm{capacity}\;\frac{mg\;VCE}{100\;g}\right)\right]$ However, we were unable to include retinol, gallocatechin, formononetin, 4-6 monomers, 7-10 monomers, and polymers in the TAC calculations. The exact source of the vitamin C equivalent (VCE) value for retinol could not be confirmed, as previously noted [32,33]. Additionally, VCE values for gallocatechin, formononetin, 4-6 monomers, 7-10 monomers, and polymers could not be found. The contents and VCE values of isoflavones were accounted for by summing the contents and VCEs of 4 types of isoflavones: daidzein, genistein, glycitein, and biochanin A. This was done despite the fact that we could extract the total isoflavones, excluding biochanin A [21]. Ultimately, the TAC values were estimated based on 41 individual components (Table 1).

### Estimating the dietary consumption of individual antioxidants, the 5 antioxidant classes, and the 7 flavonoid subclasses, as well as dietary total antioxidant capacity

#### Study population

From the KoGES, 3 general population-based cohorts were utilized: (1) the Ansan and Ansung (ASAS) study, specifically the third wave (conducted in 2005-2006); (2) the Health Examinee (HEXA) study (2004-2013); and (3) the Cardiovascular Disease Association Study (CAVAS; 2005-2011). For the ASAS study, we selected the third examination survey (2005-2006), which employed an FFQ for dietary assessment that was identical to the version used for the HEXA study and CAVAS. All 3 cohorts consisted of general population samples. However, the ASAS and CAVAS participants were community residents, while those in the HEXA study were national health examinees [3]. All participants were over 40 years old at baseline. The recruitment methods for each cohort have been described in detail elsewhere [3]. From the initial pool of 202,432 participants, we excluded individuals with implausible dietary consumption data, defined as an energy intake below the 0.5th percentile or above the 95.5th percentile, or with more than 10 missing food items (n=6,471). Consequently, a total of 195,961 participants were included in the final analysis: 7,400 from the ASAS study, 21,362 from CAVAS, and 167,199 from the HEXA study.

#### Dietary antioxidant consumption, dTAC, and energy intake based on the 106-item FFQ

Participants from the 3 population-based cohorts completed a survey assessing their average food consumption frequencies and amounts over the previous year. All surveys were administered by skilled and trained interviewers. Subsequently, the dietary intake levels of individual antioxidants, the 5 classes of antioxidants, and the 7 subclasses of flavonoids, as well as dTAC, were estimated. This was achieved by multiplying the daily frequency, portion size, and duration of consumption for seasonal foods, using the antioxidant database developed for this study. Total energy intake was calculated using the nutrient database developed by the Korean Nutrition Society, which is based on the 7th edition of the Korean Food Composition Table [34].

### Statistical analysis

The coverage of the database was defined as the proportion of food items containing data, out of the total number of foods (n=412). We compared unadjusted and age-adjusted averages of consumption for each dietary antioxidant and dTAC between men and women using the Student t-test and the general linear model, respectively. Age-adjusted and gender-adjusted dietary antioxidant intakes were analyzed according to general characteristics using the general linear model. Tukey *post-hoc* tests were used to identify significant differences between groups at a significance level of p-value< 0.05. These characteristics included cohort (ASAS, CAVAS, or HEXA), gender, age group (40s, 50s, 60s, or 70+), education level (≥ 12 years of schooling or less than that amount), smoking status (never, past, or current), drinking status (never, past, or current), regular exercise (≥ 3 times/wk and ≥ 30 min/session or not), body mass index (BMI; < 23, ≥ 23 to < 25, or ≥ 25 kg/m^2^), waist circumference (< 90 cm for men and < 85 cm for women, or ≥ 90 cm for men and ≥ 85 cm for women), and menopause status (yes or no, for women only). We also compared dietary antioxidant intakes based on the prevalence of certain chronic diseases. The prevalence of cancer and CVD was self-reported. For diabetes mellitus and hypertension, we considered both self-reported medication use and health examination data, including a fasting blood glucose level of ≥ 126 mg/dL and blood pressure (BP) of ≥ 140/90 mmHg. MetS was defined as meeting at least 3 of the following 5 criteria: (1) waist circumference ≥ 90 cm for men and ≥ 85 cm for women; (2) elevated BP, defined as systolic BP ≥ 130 mmHg and/or diastolic BP ≥ 85 mmHg, or the use of antihypertensive medication; (3) elevated fasting blood glucose level of ≥ 100 mg/dL or the use of medication for diabetes mellitus; (4) elevated triglyceride level, defined as ≥ 150 mg/dL; and (5) reduced high-density lipoprotein cholesterol level, defined as < 40 mg/dL for men and < 50 mg/dL for women. These comparisons were adjusted for age, gender, education level, smoking and drinking status, regular exercise, BMI, and all other diseases for both men and women. For women, menopause status was also considered. Furthermore, we conducted a supplementary analysis to determine the percentage contribution of each food to the intake (%) and variation (r^2^) of dTAC, the 5 antioxidant classes, and the 7 flavonoid subclasses. Statistical analyses were performed using SAS version 9.4 (SAS Institute Inc., Cary, NC, USA).

### Ethics statement

This study protocol was approved by the Hanyang University Institutional Review Board (IRB No. HYU-2020-04-003-1). All participants provided written informed consent before participating in the study.

## RESULTS

Table 1 presents a list of the 48 individual antioxidants, encompassing 5 classes of antioxidants (retinol, carotenoids, vitamin C, vitamin E, and flavonoids) and 7 flavonoid subclasses. Approximately 99% of the 412 foods analyzed contained antioxidant values, with specific coverage of 99.8% for vitamin C, 99.0% for retinol, 98.5% for vitamin E, and 92.2% for carotenoids (Table 2). The average coverage rate for flavonoids was 60.6%, with eriodictyol displaying the lowest coverage at 41.5%. In contrast, theaflavins—including theaflavin, theaflavin 3-gallate, theaflavin 3´-gallate, and theaflavin 3,3´-digallate—achieved 100% coverage due to the assignment of a logical zero value to most food groups. Likewise, all flavonoid subclasses reached 100% coverage in certain food groups, namely “Fats and oils,” “Meats and their products,” “Eggs,” “Fish and shellfish,” and “Milk and dairy products” (Supplementary Material 4).

Table 3 presents the average dietary antioxidant consumption and dTAC for both men and women. Significant differences were found in the consumption of all dietary antioxidants between genders (all p<0.001 for unadjusted and age-adjusted averages). Although men displayed a higher total energy intake than women, women demonstrated higher unadjusted and age-adjusted dTAC values (398 and 396 mg VCE/day, respectively) compared to men (380 and 385 mg VCE/day, respectively). Women consumed more retinol, carotenoids, vitamin C, total flavonoids, flavonols, flavones, flavanones, isoflavones, anthocyanins, and proanthocyanidins. Conversely, men had higher intake levels of dietary vitamin E and flavan-3-ols.

Table 4 presents the estimated dietary antioxidant intakes by cohort and general characteristics of the study population. The ASAS study participants exhibited the highest average consumption of dTAC, vitamin E, flavonols, flavonones, flavanones, flavan3-ols, and isoflavones. In contrast, the HEXA study cohort displayed the highest mean intake of retinol, carotenoids, vitamin C, anthocyanins, and proanthocyanidins. The CAVAS cohort exhibited the lowest levels of antioxidant consumption across all components. Older individuals generally demonstrated a lower intake of dTAC and antioxidants, and current smokers also tended to have reduced dTAC and antioxidant intakes. Conversely, participants with 12 or more years of education, those who engaged in regular exercise, and women who reported experiencing menopause exhibited higher dTAC and dietary antioxidant consumption. Regarding drinking status, no consistent pattern was evident in dTAC and dietary antioxidant consumption. For obesity, a higher BMI was associated with increased dTAC, but we observed no clear trend in the intake of dietary antioxidants according to BMI level. Table 5 shows dietary antioxidant intakes in relation to certain antioxidant-related diseases. For retinol, for each disease studied, intake was highest among participants without the disease. Individuals with a history of cancer tended to exhibit a higher dTAC and consume more antioxidants, such as carotenoids, vitamin C, total flavonoids, flavanones, anthocyanins, and proanthocyanidins, compared to those without cancer. However, those with CVD did not exhibit a consistent intake pattern. Relative to participants without the disease, those with diabetes mellitus generally had a lower intake of vitamin C, total flavonoids, flavones, flavanones, anthocyanins, and proanthocyanidins. Those with hypertension had a lower intake of anthocyanins only. Finally, individuals with MetS tended to have a lower intake of dTAC and all dietary antioxidants.

## DISCUSSION

In this study, we developed a comprehensive database of 48 antioxidants, including 5 classes of antioxidants, 7 subclasses of flavonoids, and TAC for 412 foods present in the recipes of the FFQ used in the KoGES. The database’s coverage of antioxidant vitamins exceeded 90% for all categories except flavonoids (60.9%), suggesting relatively high completeness. Analysis of the 3 general population cohorts yielded the following results: (1) apart from vitamin E and flavan-3-ols, women consumed most antioxidants in greater amounts than men, along with a higher TAC; (2) participants in the ASAS study had the highest intake of vitamin E, flavonols, flavonones, flavones, flavan-3-ols, and isoflavones, whereas those in the HEXA study consumed the most retinol, carotenoids, vitamin C, anthocyanins, and proanthocyanidins; and (3) intake of antioxidants and dTAC were generally lower among older participants and current smokers. Conversely, those with a higher education level, regular exercisers, and who reported experiencing menopause tended to have a higher intake of these nutrients.

This is the first study to document an expanded antioxidant database for the KoGES FFQ. To date, few studies have been conducted on the development of databases for antioxidants and TAC. These include a flavonoid database in the United States [30], 2 Korean flavonoid databases [35,36], a German flavanol database [37], a European database for a total of 437 polyphenol compounds within the European Prospective Investigation into Cancer and Nutrition [38], and databases for TAC in both the United States [15] and Korea [32]. Most of these examples derived their antioxidant values from the USDA databases [20-22] and the PhenolExplorer database [23]. Additionally, Korean databases [35,36] have utilized the Japan Functional Food Factor database [39] and the Korea Functional Food Composition Table [40], which is currently unavailable.

To ensure the quality of our database, we adhered to a standardized protocol. The overall quality and intake estimates associated with a database may be influenced by its comprehensiveness and completeness. To enhance the comprehensiveness of constituents, we expanded a nutrient database to include 48 individual antioxidants, retinol, carotenoids, vitamins C and E, flavonoids, and TAC. However, we selected only those antioxidants essential for theoretical TAC calculations [15,32,33]. Nevertheless, a need exists to further expand the database with additional research on the antioxidant capacity of a broader range of dietary antioxidants. Regarding completeness, we assessed the coverage of each constituent. Although coverage is crucial, most database development studies have not addressed this [37,38], apart from several conducted in Korea [32,35,36,41]. The antioxidant database established in the present study demonstrated relatively high coverage. Previous TAC databases have reported 99.7% and 95.3% coverage of food intake [35,41], whereas our study achieved 100% coverage. For most antioxidants, we observed over 90.0% coverage. However, flavonoids displayed a lower rate (60.6%), which was also less than the coverage noted for an earlier Korean flavonoid database (85.0%) [36]. This discrepancy may be due to the latter database’s exclusive focus on plant-based foods (n=1,549 foods from the Korea National Health and Nutrition Examination Survey [KNHANES] 2008). In comparison, a separate flavonoid database for common Korean foods had a 49% coverage rate [35] and included all food groups (n=3,193 foods from KNHANES 2007-2012). Although at 60.6%, the coverage for flavonoids in our study was not low in comparison, improving the absolute completeness of the flavonoid database remains a challenge.

Although we could not validate our findings with objective measures such as blood biomarkers in this study, we were able to indirectly assess the validity of our estimated dietary intake data [1]. We employed 2 methods for this assessment. The first approach involved comparing our estimated values with KNHANES data. The second method assessed whether the associations between dietary intakes estimated using our database and health outcomes were consistent with established evidence. In the first comparison with KNHANES, the dietary assessment method differed: 24-hour recall in KNHANES versus an FFQ in our study. Nevertheless, the daily intakes of retinol (89.8 μg/day for men and 92.2 μg/day for women) and vitamin C (53 mg/day for men and 62.7 mg/day for women) in our study were comparable to the retinol levels reported in KNHANES 2007-2012 (93.6 μg/day for individuals aged 50-64 years; 61.8 μg/day for those aged 65-74 years) [42] and the vitamin C consumption data in KNHANES 2016-2018 (60.6 mg/day for all ages) [43]. Carotenoid intake (7.51 mg/day for men and 8.79 mg/day for women) was also similar to that in KNHANES 2007-2012 (9.3 mg/day for individuals aged 50-64 years and 7.5 mg/day for those aged 65-74 years) [42]. The dTAC consumption in the present study was 385 mg VCE/day for men and 396 mg VCE/day for women, aligning with the findings of a previous Korean study using KNHANES 2007-2012 data (384.7 mg VCE/day for individuals aged 19 years and older) [32]. However, the estimated daily intake of total flavonoids (215 mg/day for men and 236 mg/day for women) was lower than that for participants aged 19 years and older in KNHANES 2007-2012 (318.0 mg/day) [35]. Notably, the database used in the latter study was based on the Korea Functional Food Composition Table [40], which is currently unavailable due to issues with sample pre-processing and analysis methods. Given the potential for overestimation of their calculated intakes, their results may not be directly comparable to ours. For the second method of indirect validation, our analyses—grounded in well-supported hypotheses regarding possible mechanisms—uncovered significant inverse associations between antioxidant intakes, particularly flavonoids, and the risk of hypertension and MetS. These intakes were calculated using the same databases as the present study [44,45].

The low daily intake of antioxidants and dTAC among the CAVAS participants, relative to the other cohorts, could be attributed to the older average age of its members. A recent study analyzing 2013-2018 KNHANES data revealed that only 35.47% of elderly Korean individuals (aged ≥ 65 years) met the World Health Organization’s recommended fruit and vegetable intake level of 400 g/day [46]. This may also account for the observed trend of decreased dietary antioxidant consumption, including dTAC, with advancing age in our study. Of the flavonoids, only anthocyanins and proanthocyanidins were consumed in greater amounts in the HEXA study, which primarily included urban areas, than in the other cohorts. This could be due to the higher consumption of specific foods like grapes, grape juice, apples, and apple juice, which heavily contributed to the variation (r^2^ > 80%) and intake (> 30%). These fruits are also key sources of vitamin C and carotenoids, causing the HEXA study to report the highest intake of these nutrients. In the present study, more highly educated participants (those with 12 or more years of education) displayed greater dTAC and antioxidant intakes than the less educated group, while current smokers had lower intakes than those with other smoking statuses. This aligns with previous findings indicating lower consumption of fruits and vegetables among current smokers and those with lower education levels [47]. Additionally, we observed that regular exercisers consumed more fruits and vegetables than those who did not exercise regularly, a finding supported by a recent study that found a positive association between physical activity and fruit and vegetable consumption [48]. Due to the potential for reverse causation, differences in antioxidant intake according to disease prevalence should not be interpreted as indicative of cause-and-effect relationships. The higher dTAC and intakes of most dietary antioxidants among patients with cancer may have been influenced by substantial research suggesting the protective effects of dietary antioxidants against cancer [13]. Conversely, the results related to diabetes mellitus may be impacted by reverse causation in the opposite direction, as individuals with diabetes may reduce their fruit intake, with fruits like oranges, grapes, strawberries, apples, and tangerines being major antioxidant contributors in this study (Supplementary Material 5). Although the trends for diabetes mellitus and hypertension in the present study did not closely align with the prospective relationships identified in the CAVAS [44,49], and the consistently higher retinol intake among non-disease participants in this study remains unexplained, those with MetS did tend to consume lower amounts of most dietary antioxidants. This observation supports the prospective associations between antioxidants and MetS identified using the same database in the CAVAS [45].

Some limitations should be considered when interpreting our findings. First, although our antioxidant database demonstrated relatively high coverage compared to previous databases, it retains room for improvement in the completeness of certain flavonoids, such as eriodictyol, which exhibited a coverage of 41.5%. Second, to ensure the quality of the database, we exclusively used source databases from government and authorized institutions. However, a reliance on foreign flavonoid databases—which originate from countries with different food cultivation, growing, and production conditions than Korea—represents a limitation of our database, despite most nutrient databases being developed based on the USDA food composition sources. Third, we were unable to compare the antioxidant intake and capacity derived from our database with other objective measures, such as corresponding biomarkers. Despite these limitations, this is the first antioxidant database to include antioxidant capacity for the KoGES FFQ, which demonstrates the potential for standardized procedures to expand the food composition of that instrument. Furthermore, considering the comparable estimated values to the KNHANES and the significant and suggested results obtained in previous studies [44,45], the validity of our database may be considered acceptable.

In conclusion, we have constructed a comprehensive antioxidant database for the KoGES, a representative cohort widely utilized in Korea. This database represents a valuable and practical resource for future research aimed at exploring the associations between dietary antioxidant intake and various health outcomes.

## Figures and Tables

**Table 1.** All 48 individual antioxidants, 5 classes of antioxidants, and 7 subclasses of flavonoids in the antioxidant database for the estimation of dietary total antioxidant capacity

Classes of antioxidants (5)	Subclasses of flavonoids (7)	Individual antioxidants (48)	
		Components	No. of components^1^
Retinol		Retinol^2^	1 (0)
Vitamin C		Vitamin C (ascorbic acid)	1 (1)
Vitamin E		Alpha-tocopherol, beta-tocopherol, gamma-tocopherol, delta-tocopherol	4 (4)
Carotenoids		Alpha-carotene, beta-carotene, lycopene, beta-cryptoxanthin, lutein and zeaxanthin^2^	5 (5)
Flavonoids	Flavonols	Quercetin, kaempferol, myricetin, isorhamnetin	4 (4)
	Flavones	Luteolin, apigenin	2 (2)
	Flavanones	Hesperetin, naringenin, eriodictyol	3 (3)
	Flavan-3-ols	Catechin, epicatechin, epigallocatechin, epicatechin 3-gallate, epigallocatechin 3-gallate, gallocatechin2, theaflavin, thearubigin, theaflavin 3-gallate, theaflavin 3´-gallate, theaflavin 3,3´-digallate	11 (10)
	Anthocyanins	Cyanidin, delphinidin, malvidin, pelargonidin, peonidin, petunidin	6 (6)
	Isoflavones	Total isoflavones^2^, daidzein, genistein, glycitein, biochanin, formononetin^2^	6 (4)
	Proanthocyanidins	Dimers, trimers, 4-6 monomers^2^, 7-10 monomers^2^, polymers^2^	5 (2)

1 Values are presented as total number of components included in the database, with the number of components used to estimate dietary total antioxidant capacity in parentheses.

2 Not included in the estimation of dietary total antioxidant capacity.

**Table 2.** Number of foods used to develop the antioxidant database and the coverage of each component by original database and imputation method

Foods	Database				Imputation method				Total no. of foods	Coverage (%)
	KMFDS	USDA	INRA	JMEXT	Moisture conversion	Calculation of recipe	Similar food items	Zero values		
Retinol (μg)^1^	362	28^2^	-	7	5	-	6	-	408	99.0
Carotenoids (mg)									378	92.2
Alpha-carotene (μg)	-	186^2^	-	70	11	19	31	61		91.7
Beta-carotene (μg)	361	25^2^	-	5	8	-	10	3	412	100
Lycopene (μg)	-	185^2^	-	68	17	19	33	61	383	93.0
Beta cryptoxanthin (μg)	-	186^2^	-	68	11	19	33	61	378	91.7
Lutein and zeaxanthin (μg)	-	183^2^	-	-	17	18	49	82	349	84.7
Vitamin C (mg)	368	26^2^	-	7	6	-	4	-	411	99.8
Vitamin E (mg)										98.5
Alpha-tocopherol (mg)	360	24^2^	-	9	7	-	8	-	408	99.0
Beta-tocopherol (mg)	360	9^2^	-	16	7	-	13	-	405	98.3
Gamma-tocopherol (mg)	360	9^2^	-	16	7	-	13	-	405	98.3
Delta-tocopherol (mg)	360	9^2^	-	16	7	-	13	-	405	98.3
Total flavonoids (mg)										60.6
Flavonols (mg)										60.3
Quercetin (mg)	-	63^3^	6	-	8	14	20	161	272	66.0
Kaempferol (mg)	-	56^3^	9	-	9	14	19	161	268	65.0
Myricetin (mg)	-	50^3^	8	-	8	12	20	161	259	62.9
Isorhamnetin (mg)	-	12^3^	11	-	1	5	3	162	194	47.1
Flavones (mg)										47.7
Luteolin (mg)	-	19^3^	8	-	-	3	7	162	199	48.3
Apigenin (mg)	-	21^3^	1	-	-	3	7	162	194	47.1
Flavanones (mg)										45.6
Hesperetin (mg)	-	19^3^	8	-	-	3	7	162	199	48.3
Naringenin (mg)	-	21^3^	1	-	-	3	7	162	194	47.1
Eriodictyol (mg)	-	1^3^	5	-	-	-	3	162	171	41.5
Flavan-3-ols (mg)										75.5
Catechin (mg)	-	36^3^	6	-	2	5	19	161	229	55.6
Epicatechin (mg)	-	37^3^	6	-	2	9	18	161	233	56.6
Epigallocatechin (mg)	-	33^3^	4	-	3	5	20	161	226	54.9
Epicatechin 3-gallate (mg)	-	33^3^	4	-	3	4	20	161	225	54.6
Epigallocatechin 3-gallate (mg)	-	33^3^	4	-	3	5	18	161	224	54.4
Gallocatechin (mg)^1^	-	33	4	-	3	5	20	161	226	54.9
Theaflavin (mg)	-	1^3^	-	-	-	-	-	411	412	100
Thearubigin (mg)	-	1^3^	-	-	-	-	-	411	412	100
Theaflavin 3-gallate (mg)	-	1^3^	-	-	-	-	-	411	412	100
Theaflavin 3´-gallate (mg)	-	1^3^	-	-	-	-	-	411	412	100
Theaflavin 3,3´-digallate (mg)	-	1^3^	-	-	-	-	-	411	412	100
Anthocyanins (mg)										71.4
Cyanidin (mg)	-	21^3^	4	-	-	3	7	264	299	72.6
Delphinidin (mg)	-	16^3^	2	-	-	4	6	267	295	71.6
Malvidin (mg)	-	14^3^	2	-	-	4	6	267	293	71.1
Pelargonidin (mg)	-	17^3^	-	-	-	3	6	267	293	71.1
Peonidin (mg)	-	15^3^	2	-	-	3	6	267	293	71.1
Petunidin (mg)	-	14^3^	2	-	-	4	6	267	293	71.1
Isoflavones (mg)										60.9
Daidzein (mg)	-	76^4^	1	-	9	25	28	154	293	71.1
Genistein (mg)	-	76^4^	1	-	9	25	28	154	293	71.1
Glycitein (mg)	-	34^4^	1	-	6	17	9	155	222	53.9
Biochanin (mg)	-	10^4^	-	-	7	14	3	161	195	47.3
Formononetin (mg)^1^	-	24	-	-	8	17	10	159	218	52.9
Proanthocyanidins (mg)										63.1
Dimers (mg)	-	52^5^	5	-	6	24	21	161	269	65.3
Trimers (mg)	-	50^5^	4	-	7	24	20	161	266	64.6
4-6 monomers (mg)^1^	-	45^5^	2	-	6	23	20	161	257	62.4
7-10 monomers (mg)^1^	-	45^5^	2	-	5	23	20	161	256	62.1
Polymers (mg)^1^	-	43^5^	2	-	5	23	18	161	252	61.2

KMFDS, Food and Nutrient Database of the Korea Ministry of Food and Drug Safety (accessed on July 17, 2020); USDA, United States Department of Agriculture; INRA, Phenol-Explorer 3.6 of Institut National de la Recherche Agronomique (accessed on May 25, 2020); JMEXT, Standard Tables of Food Composition in Japan (2015; 7th edition) of the Ministry of Education, Culture, Sports, Science and Technology Japan (accessed on May 6, 2020).

1 These components (retinol; gallocatechin from the flavan-3-ols; formononetin from the isoflavones; and 4-6 monomers, 7-10 monomers, and polymers from the proanthocyanidins) were not included in the estimation of dietary total antioxidant capacity.

2 USDA National Nutrient Database for Standard Reference, release 28 (2015).

3 USDA Database for the Flavonoid Content of Selected Foods, release 3.3 (2018).

4 USDA Database for the Isoflavone Content of Selected Foods, release 2.1.

5 USDA Database for the Proanthocyanidin Content of Selected Foods, release 2.1 (accessed on May 25, 2020).

**Table 3.** Unadjusted and age-adjusted dTAC and daily consumption of antioxidants for participants in all 3 general population cohorts, KoGES (n=195,961)^1^

Component of antioxidant database	Total	Unadjusted daily intake (mean±SD)		Age-adjusted daily intake average (mean±SE)	
		Men	Women	Men	Women
Total (n)	195,961	69,090	126,871	69,090	126,871
Total energy intake (kcal/day)	1,678±516	1,779±2	1,624±1	1,787±2	1,619±1
dTAC (mg VCE/day)	392±445	380±2	398±1	385±2	396±1
Five classes of antioxidants					
Retinol (μg/day)	91.3±86.2	88.6±0.3	92.9±0.2	89.8±0.3	92.2±0.2
Carotenoids (mg/day)	8.34±7.50	7.48±0.03	8.8±0.02	7.51±0.03	8.79±0.02
Alpha-carotene (μg/day)	360±563	318±2	382±2	320±2	381±2
Beta-carotene (μg/day)	2,105±1,644	2,033±6	2,144±5	2,041±6	2,139±5
Lycopene (μg/day)	3,461±5,171	2,912±20	3,760±15	2,921±20	3,755±15
Beta cryptoxanthin (μg/day)	395±467	329±2	431±1	330±2	430±1
Lutein and zeaxanthin (μg/day)	2,016±2,008	1,887±8	2,086±6	1,899±8	2,080±6
Vitamin C (mg/day)	59.3±42.4	52.6±0.2	62.9±0.1	53.0±0.2	62.7±0.1
Vitamin E (mg/day)	5.49±3.70	5.65±0.01	5.40±0.01	5.70±0.01	5.37±0.01
Alpha-tocopherol (mg/day)	1.92±1.22	2.02±0.00	1.87±0.00	2.05±0.00	1.86±0.00
Beta-tocopherol (mg/day)	0.09±0.08	0.10±0.00	0.08±0.00	0.11±0.00	0.08±0.00
Gamma-tocopherol (mg/day)	2.51±1.95	2.54±0.01	2.49±0.01	2.55±0.01	2.48±0.01
Delta-tocopherol (mg/day)	0.97±0.85	0.99±0.00	0.96±0.00	1.00±0.00	0.96±0.00
Total flavonoids (mg/day)	228±224	213±1	237±1	215±1	236±1
Seven subclasses of flavonoids					
Flavonols (mg/day)	22.1±18.5	21.6±0.1	22.3±0.1	21.8±0.1	22.2±0.1
Quercetin (mg/day)	14.0±13.1	13.0±0.1	14.5±0.0	13.1±0.1	14.4±0.0
Kaempferol (mg/day)	5.86±5.78	6.29±0.02	5.63±0.02	6.32±0.02	5.62±0.02
Myricetin (mg/day)	1.40±1.40	1.46±0.01	1.37±0.00	1.48±0.01	1.36±0.00
Isorhamnetin (mg/day)	0.83±0.88	0.85±0.00	0.81±0.00	0.85±0.00	0.81±0.00
Flavones (mg/day)	2.00±1.49	1.85±0.01	2.09±0.00	1.87±0.01	2.08±0.00
Luteolin (mg/day)	1.16±0.83	1.12±0.00	1.18±0.00	1.12±0.00	1.18±0.00
Apigenin (mg/day)	0.85±0.92	0.74±0.00	0.91±0.00	0.75±0.00	0.90±0.00
Flavanones (mg/day)	9.74±13.30	7.70±0.05	10.9±0.00	7.83±0.05	10.8±0.00
Hesperetin (mg/day)	4.43±7.51	3.61±0.03	4.88±0.02	3.68±0.03	4.84±0.02
Naringenin (mg/day)	5.29±6.84	4.07±0.03	5.95±0.02	4.13±0.03	5.91±0.02
Eriodictyol (mg/day)	0.02±0.04	0.02±0.00	0.03±0.00	0.02±0.00	0.03±0.00
Flavan-3-ols (mg/day)	85.1±161.00	87.9±1.00	83.5±0.00	89.6±1.00	82.6±0.00
Catechin (mg/day)	6.11±6.99	5.54±0.03	6.42±0.02	5.62±0.03	6.37±0.02
Epicatechin (mg/day)	9.94±11.73	9.60±0.04	10.20±0.00	9.70±0.04	10.10±0.00
Epigallocatechin (mg/day)	17.3±35.2	18.2±0.1	16.8±0.1	18.6±0.1	16.6±0.1
Epicatechin 3-gallate (mg/day)	10.5±21.6	11.0±0.1	10.2±0.1	11.2±0.1	10.1±0.1
Epigallocatechin 3-gallate (mg/day)	39.7±84.4	42.0±0.3	38.4±0.2	42.8±0.3	38.0±0.2
Gallocatechin (mg/day)	0.90±1.85	0.94±0.01	0.87±0.01	0.96±0.01	0.86±0.01
Theaflavin (mg/day)	0.03±0.06	0.03±0.00	0.03±0.00	0.03±0.00	0.03±0.00
Thearubigin (mg/day)	0.61±1.30	0.64±0.00	0.59±0.00	0.66±0.00	0.58±0.00
Theaflavin 3-gallate (mg/day)^2^	0.00	0.00	0.00	0.00	0.00
Theaflavin 3´-gallate (mg/day)	0.01±0.01	0.01±0.00	0.01±0.00	0.01±0.00	0.01±0.00
Theaflavin 3,3´-digallate (mg/day)	0.01±0.01	0.01±0.00	0.01±0.00	0.01±0.00	0.01±0.00
Anthocyanins (mg/day)	10.50±12.70	8.46±0.05	11.60±0.00	8.55±0.05	11.50±0.00
Cyanidin (mg/day)	2.09±2.39	1.72±0.01	2.29±0.01	1.73±0.01	2.29±0.01
Delphinidin (mg/day)	0.33±0.52	0.26±0.00	0.37±0.00	0.27±0.00	0.37±0.00
Malvidin (mg/day)	4.58±7.70	3.59±0.03	5.12±0.02	3.63±0.03	5.10±0.02
Pelargonidin (mg/day)	2.67±3.61	2.27±0.01	2.88±0.01	2.29±0.01	2.87±0.01
Peonidin (mg/day)	0.54±0.88	0.43±0.00	0.61±0.00	0.43±0.00	0.61±0.00
Petunidin (mg/day)	0.25±0.41	0.20±0.00	0.28±0.00	0.20±0.00	0.28±0.00
Isoflavones (mg/day)	20.4±17.9	20.3±0.1	20.5±0.1	20.2±0.1	20.5±0.1
Daidzein (mg/day)	8.58±7.49	8.53±0.03	8.61±0.02	8.52±0.03	8.62±0.02
Genistein (mg/day)	9.80±8.73	9.69±0.03	9.86±0.02	9.68±0.03	9.87±0.02
Glycitein (mg/day)	1.89±1.71	1.86±0.01	1.90±0.00	1.85±0.01	1.90±0.00
Biochanin (mg/day)	0.01±0.02	0.01±0.00	0.01±0.00	0.01±0.00	0.01±0.00
Formononetin (mg/day)	0.16±0.13	0.18±0.00	0.14±0.00	0.18±0.00	0.14±0.00
Proanthocyanidins (mg/day)	78.6±85.2	65.0±0.3	86.1±0.2	65.5±0.3	85.8±0.2
Dimers (mg/day)	12.2±12.4	10.5±0.1	13.2±0.0	10.6±0.1	13.1±0.0
Trimers (mg/day)	6.57±6.02	5.69±0.02	7.04±0.02	5.72±0.02	7.03±0.02
4-6 monomers (mg/day)	17.7±20.1	14.7±0.1	19.4±0.1	14.8±0.1	19.3±0.1
7-10 monomers (mg/day)	13.3±17.4	10.7±0.1	14.7±0.1	10.8±0.1	14.6±0.1
Polymers (mg/day)	28.9±31.5	23.5±0.1	31.8±0.1	23.7±0.1	31.7±0.1

dTAC, dietary total antioxidant capacity; KoGES, Korean Genome and Epidemiology Study; VCE, vitamin C equivalent.

1 All p-values for comparisons by gender, obtained via Student t-test for unadjusted consumption and the general linear model for age-adjusted consumption, were <0.001.

2 The content was zero for all food items in the food frequency questionnaire.

**Table 4.** Age- and gender-adjusted dTAC, along with daily consumption of 5 antioxidant classes and 7 flavonoid subclasses, by general characteristics

Characteristics	%	dTAC (mg VCE/day)	p-value^1^	Five classes of antioxidants									
				Retinol (μg/day)	p-value^1^	Carotenoids (mg/day)	p-value^1^	Vitamin C (mg/day)	p-value^1^	Vitamin E (mg/day)	p-value^1^	Total flavonoids (mg/day)	p-value^1^
Total (n=195,961)													
Cohort study													
ASAS	3.8	469±5.13^a^	<0.001	83.9±0.99^a^	<0.001	7.62±0.09^a^	<0.001	53.9±0.49^a^	<0.001	6.23±0.04^a^	<0.001	261±2.58^a^	<0.001
CAVAS	10.9	341±3.09^b^		71.6±0.60^b^		7.04±0.05^b^		49.6±0.29^b^		5.00±0.03^b^		190±1.56^b^	
HEXA	85.3	393±1.13^c^		93.8±0.22^b^		8.32±0.02^c^		59.1±0.11^c^		5.57±0.01^c^		228±0.57^c^	
Gender													
Men	35.3	385±1.68	<0.001	89.8±0.33	<0.001	7.51±0.03	<0.001	53.0±0.16	<0.001	5.70±0.01	<0.001	215±0.85	<0.001
Women	64.7	396±1.24		92.2±0.24		8.79±0.02		62.7±0.12		5.37±0.01		236±0.63	
Age (yr)													
40-49	34.6	440±1.73^a^	<0.001	102±0.34^a^	<0.001	8.27±0.03^a^	<0.001	60.8±0.16^a^	<0.001	6.02±0.01^a^	<0.001	248±0.87^a^	<0.001
50-59	37.5	399±1.67^b^		91.7±0.32^b^		8.47±0.03^b^		59.5±0.16^b^		5.55±0.01^b^		232±0.84^b^	
60-69	24.2	328±2.04^c^		78.7±0.39^c^		7.80±0.03^c^		53.6±0.19^c^		5.02±0.02^c^		196±1.03^c^	
70+	3.7	248±5.17^d^		58.2±1.00^d^		6.17±0.09^d^		41.6±0.49^d^		4.28±0.04^d^		148±2.60^d^	
Higher education^2^													
Yes	60.8	424±1.34	<0.001	98.6±0.26	<0.001	8.68±0.02	<0.001	62.1±0.13	<0.001	5.84±0.01	<0.001	247±0.67	<0.001
No	39.2	331±1.79		77.8±0.35		7.24±0.03		50.6±0.17		5.02±0.01		188±0.90	
Smoking status													
Never smoker	72.1	398±1.62^a^	<0.001	91.4±0.31^a^	<0.001	8.40±0.03^a^	<0.001	59.4±0.15^a^	<0.001	5.52±0.01^a^	0.006	231±0.82^a^	<0.001
Past smoker	14.9	401±2.92^a^		91.9±0.57^a^		8.07±0.05^b^		57.6±0.28^b^		5.60±0.02^b^		231±1.47^a^	
Current smoker	13.0	350±3.02^b^		88.4±0.58^b^		7.34±0.05^c^		52.4±0.29^c^		5.51±0.03^a^		197±1.52^b^	
Drinking status													
Never drinker	50.7	381±1.65^a^	<0.001	88.8±0.32^a^	<0.001	8.33±0.03^a^	<0.001	59.0±0.16^a^	<0.001	5.51±0.01^a^	<0.001	224±0.83^a^	0.001
Past drinker	4.3	401±4.86^b^		90.8±0.94^a^,^b^		8.27±0.08^a^		59.2±0.46^a^		5.73±0.04^b^		234±2.45^b^	
Current drinker	45.1	397±1.51^b^		92.8±0.29^b^		7.97±0.03^b^		56.7±0.14^b^		5.54±0.01^a^		226±0.76^a^	
Regular exercise^3^													
Yes	34.3	448±1.74	<0.001	101±0.34	<0.001	9.14±0.03	<0.001	64.0±0.17	<0.001	5.93±0.01	<0.001	260±0.88	<0.001
No	65.6	358±1.28		85.0±0.25		7.59±0.02		54.4±0.12		5.31±0.01		206±0.64	
BMI (kg/m^2^)													
<23	38.4	368±1.68^a^	<0.001	91.2±0.32^a^,^b^	0.009	8.06±0.03^a^	<0.001	57.5±0.16^a^	<0.001	5.47±0.01^a^	<0.001	217±0.84^a^	<0.001
≥23 to <25	27.6	399±1.92^b^		91.7±0.37^a^		8.24±0.03^b^		58.5±0.18^b^		5.54±0.02^b^		230±0.97^b^	
≥25	34.1	406±1.72^c^		90.2±0.33^b^		8.17±0.03^b^		57.7±0.16^a^		5.60±0.01^c^		230±0.87^b^	
Waist circumference (cm)													
<90 for men/85 for women	73.1	386±1.22	<0.001	91.6±0.24	<0.001	8.17±0.02	0.003	58.3±0.12	<0.001	5.52±0.01	0.137	225±0.62	0.577
≥90 for men/85 for women	26.9	398±1.96		89.0±0.38		8.06±0.03		56.6±0.19		5.55±0.02		225±0.99	
Menopause (for women)													
Yes	64.4	404±1.81	<0.001	93.9±0.36	<0.001	9.07±0.03	<0.001	64.5±0.18	<0.001	5.44±0.02	<0.001	241±0.93	<0.001
No	35.6	387±2.69		90.8±0.53		8.32±0.05		60.1±0.27		5.32±0.02		229±1.38	

Characteristics	%	Seven subclasses of flavonoids (mg/day)													
		Flavonols	p-value^1^	Flavones	p-value^1^	Flavanones	p-value^1^	Flavan-3-ols	p-value^1^	Anthocyanins	p-value^1^	Isoflavones	p-value^1^	Proanthocyanidins	p-value^1^
Total (n=195,961)															
Cohort study															
ASAS	3.8	23.1±0.21^a^	<0.001	2.05±0.02^a^	<0.001	9.78±0.15^a^	<0.001	118±1.86^a^	<0.001	9.54±0.15^a^	<0.001	28.4±0.21^a^	<0.001	70.1±0.98^a^	<0.001
CAVAS	10.9	19.1±0.13^b^		1.91±0.01^b^		8.85±0.09^b^		76.5±1.12^b^		8.31±0.09^b^		19.6±0.13^b^		55.9±0.59^b^	
HEXA	85.3	22.3±0.05^c^		1.98±0.00^c^		9.34±0.03^c^		85.8±0.41^c^		10.3±0.03^c^		20.1±0.05^c^		78.5±0.22^c^	
Gender															
Men	35.3	21.8±0.07	<0.001	1.87±0.01	<0.001	7.80±0.05	<0.001	89.6±0.61	<0.001	8.50±0.05	<0.001	20.2±0.07	0.001	65.5±0.32	<0.001
Women	64.7	22.2±0.05		2.08±0.00		10.8±0.04		82.6±0.45		11.5±0.04		20.5±0.05		85.8±0.24	
Age (yr)															
40-49	34.6	22.9±0.07^a^	<0.001	2.12±0.01^a^	<0.001	10.6±0.05^a^	<0.001	104±0.63^a^	<0.001	10.7±0.05^a^	<0.001	19.8±0.07^a^	<0.001	78.2±0.33^a^	<0.001
50-59	37.5	22.7±0.07^a^		2.01±0.01^b^		9.40±0.05^b^		87.3±0.60^b^		10.5±0.05^b^		20.9±0.07^b^		78.7±0.32^a^	
60-69	24.2	20.4±0.09^b^		1.79±0.01^c^		7.84±0.06^c^		65.3±0.74^c^		8.90±0.06^c^		20.7±0.08^b^		71.1±0.39^b^	
≥70	3.7	16.2±0.22^c^		1.44±0.02^d^		5.82±0.15^d^		47.1±1.88^d^		6.40±0.15^d^		19.3±0.21^a^		51.9±0.99^c^	
Higher education^2^															
Yes	60.8	22.8±0.06	<0.001	2.05±0.00	<0.001	10.2±0.04	<0.001	95.1±0.49	<0.001	11.0±0.04	<0.001	20.8±0.05	<0.001	85.4±0.25	<0.001
No	39.2	20.6±0.07		1.85±0.01		7.80±0.05		70.5±0.65		8.40±0.05		19.6±0.07		59.0±0.34	
Smoking status															
Never smoker	72.1	22.1±0.07^a^	0.019	1.99±0.01^a^	<0.001	9.74±0.05^a^	<0.001	87.4±0.59^a^	<0.001	10.5±0.05^a^	<0.001	20.4±0.07^a^	<0.001	79.1±0.31^a^	<0.001
Past smoker	14.9	22.0±0.12^a^,^b^		1.97±0.01^a^		9.18±0.09^b^		90.3±1.06^a^		9.98±0.08^a^		20.6±0.12^a^		77.4±0.56^b^	
Current smoker	13.0	21.6±0.13^b^		1.90±0.01^b^		7.88±0.09^c^		76.3±1.10^b^		8.45±0.09^c^		19.9±0.12^b^		61.2±0.58^c^	
Drinking status															
Never drinker	50.7	21.7±0.07^a^	<0.001	1.95±0.01^a^	<0.001	9.59±0.05^a^	<0.001	81.2±0.60^a^	<0.001	10.5±0.05^a^	<0.001	20.5±0.07^a^	<0.001	78.8±0.32^a^	<0.001
Past drinker	4.3	22.0±0.20^a^,^b^		1.99±0.02^a^,^b^		9.82±0.14^a^		88.1±1.76^b^		10.5±0.14^a^		21.5±0.20^b^		80.0±0.93^a^	
Current drinker	45.1	22.3±0.06^b^		1.99±0.01^b^		9.01±0.04^b^		90.1±0.55^b^		9.62±0.04^b^		20.1±0.06^c^		72.5±0.29^b^	
Regular exercise^3^															
Yes	34.3	24.1±0.07	<0.001	2.13±0.01	<0.001	10.3±0.05	<0.001	102±0.63	<0.001	11.4±0.05	<0.001	22.1±0.07	<0.001	88.9±0.33	<0.001
No	65.6	20.8±0.05		1.89±0.00		8.78±0.04		77.6±0.46		9.30±0.04		19.5±0.05		68.6±0.24	
BMI (kg/m^2^)															
<23	38.4	21.1±0.07^a^	<0.001	1.90±0.01^a^	<0.001	9.40±0.05^a^	<0.001	77.5±0.61^a^	<0.001	10.2±0.05^a^	<0.001	20.1±0.07^a^	<0.001	77.1±0.32^a^	<0.001
≥23 to <25	27.6	22.2±0.08^b^		1.99±0.01^b^		9.40±0.06^a^		89.0±0.70^b^		10.2±0.05^a^		20.5±0.08^b^		76.9±0.37^a^	
≥25	34.1	22.7±0.07^c^		2.03±0.01^c^		9.12±0.05^b^		92.6±0.63^c^		9.77±0.05^b^		20.6±0.07^b^		73.1±0.33^b^	
Waist circumference (cm)															
<90 for men/85 for women	73.1	21.8±0.05	<0.001	1.95±0.00	<0.001	9.40±0.04	<0.001	83.8±0.44	<0.001	10.2±0.03	<0.001	20.4±0.05	0.812	77.7±0.23	<0.001
≥90 for men/85 for women	26.9	22.5±0.08		2.01±0.01		8.96±0.06		91.0±0.71		9.50±0.06		20.4±0.08		70.2±0.38	
Menopause (for women)															
Yes	64.4	22.8±0.08	<0.001	2.12±0.01	<0.001	11.0±0.06	<0.001	83.8±0.65	0.385	11.9±0.06	<0.001	21.0±0.07	<0.001	88.5±0.37	<0.001
No	35.6	21.2±0.12		2.02±0.01		10.6±0.09		82.6±0.96		11.0±0.08		19.7±0.11		81.8±0.55	

Values are presented as mean±standard error; Mean values with different superscripts (a, b, c) within a row represent significant differences among the exposure groups on the Tukey multiple comparison test. dTAC, dietary total antioxidant capacity; BMI, body mass index; ASAS, Ansan and Ansung study; HEXA, Health Examinee study; CAVAS, Cardiovascular Disease Association Study.

1 p-values were obtained with the general linear model after adjusting for age and gender; The age-adjusted average for gender and gender-adjusted average for age are presented.

2 A “yes” response was defined as ≥12 years of schooling.

3 A “yes” response was defined as exercising ≥3 times/wk for ≥30 min/session, while a “no” response indicated exercising <3 times/wk and/or <30 min/session.

**Table 5.** dTAC, along with daily consumption of 5 antioxidant classes and 7 flavonoid subclasses, by disease status^1^

Characteristics	Prevalence (%)	dTAC (mg VCE/day)	p-value^1^	Five classes of antioxidants									
				Retinol (μg/day)	p-value^1^	Carotenoids (mg/day)	p-value^1^	Vitamin C (mg/day)	p-value^1^	Vitamin E (mg/day)	p-value^1^	Total flavonoids (mg/day)	p-value^1^
n=195,961													
Cancer													
Yes	3.2	429±10.1	0.021	89.0±1.99	<0.001	9.12±0.18	<0.001	63.2±1.01	<0.001	5.71±0.08	0.258	249±5.18	<0.001
No	96.8	412±7.30		95.2±1.43		8.44±0.13		59.1±0.73		5.64±0.06		234±3.74	
Cardiovascular disease													
Yes	4.1	424±10.6	0.450	89.8±2.08	0.004	8.87±0.19	0.216	61.3±1.05	0.665	5.64±0.09	0.314	242±5.41	0.629
No	95.9	417±7.17		94.4±1.41		8.68±0.13		61.0±0.72		5.71±0.06		240±3.67	
Diabetes mellitus													
Yes	8.6	419±9.22	0.580	90.5±1.81	0.007	8.76±0.16	0.762	59.2±0.92	<0.001	5.66±0.08	0.739	236±4.73	0.001
No	91.4	422±7.82		93.6±1.54		8.79±0.14		63.0±0.78		5.68±0.06		246±4.00	
Hypertension													
Yes	29.8	420±8.30	0.728	90.1±1.63	<0.001	8.74±0.15	0.252	60.8±0.83	0.109	5.67±0.07	0.991	240±4.25	0.313
No	70.2	421±8.14		94.0±1.60		8.81±0.14		61.4±0.81		5.67±0.07		242±4.17	
Metabolic syndrome													
Yes	23.9	410±8.37	<0.001	88.2±1.65	<0.001	8.61±0.15	<0.001	60.1±0.84	<0.001	5.59±0.07	<0.001	235±4.29	<0.001
No	76.1	430±8.21		96.0±1.61		8.94±0.15		62.2±0.82		5.75±0.07		247±4.21	

Characteristics	Prevalence (%)	Seven subclasses of flavonoids (mg/day)													
		Flavonols	p-value^1^	Flavones	p-value^1^	Flavanones	p-value^1^	Flavan-3-ols	p-value^1^	Anthocyanins	p-value^1^	Isoflavones	p-value^1^	Proanthocyanidins	p-value^1^
Cancer															
Yes	3.2	23.5±0.43	0.393	2.08±0.04	0.126	10.2±0.32	0.001	95.1±3.64	0.458	10.9±0.31	<0.001	21.4±0.41	0.107	85.7±2.09	<0.001
No	96.8	23.2±0.31		2.04±0.03		9.39±0.23		93.2±2.63		9.87±0.23		21.0±0.30		74.8±1.51	
Cardiovascular disease															
Yes	4.1	23.7±0.45	0.101	2.06±0.04	0.827	9.87±0.34	0.562	95.1±3.80	0.537	10.6±0.33	0.116	21.1±0.43	0.692	79.9±2.18	0.639
No	95.9	23.1±0.31		2.06±0.02		9.71±0.23		93.2±2.58		10.2±0.22		21.3±0.29		80.7±1.48	
Diabetes mellitus															
Yes	8.6	23.4±0.40	0.964	2.01±0.03	<0.001	9.00±0.30 <0.001		95.8±3.32	0.120	9.47±0.29	<0.001	21.3±0.37	0.326	75.2±1.91	<0.001
No	91.4	23.4±0.34		2.11±0.03		10.6±0.25		92.5±2.81		11.3±0.24		21.1±0.32		85.3±1.61	
Hypertension															
Yes	29.8	23.4±0.36	0.662	2.06±0.03	0.959	9.70±0.27	0.110	94.2±2.99	0.899	10.3±0.26	0.010	21.2±0.34	0.820	79.5±1.71	0.033
No	70.2	23.3±0.35		2.06±0.03		9.88±0.26		94.1±2.93		10.5±0.25		21.2±0.33		81.0±1.68	
Metabolic syndrome															
Yes	23.9	23.1±0.36	0.002	2.03±0.03	0.001	9.61±0.27	0.007	91.5±3.01	<0.001	10.2±0.26	<0.001	21.0±0.34	0.005	77.7±1.73	<0.001
No	76.1	23.6±0.35		2.08±0.03		9.97±0.26		96.9±2.95		10.6±0.25		21.4±0.33		82.9±1.70	

Values are presented as mean±standard error. dTAC, dietary total antioxidant capacity.

1 p-values were obtained with the general linear model after adjusting for gender, age (years), education level (≥12 years of schooling or less), smoking status (never/past/current), drinking status (never/past/current), regular exercise (≥3 times/wk and ≥30 min/session or not), body mass index (kg/m^2^), menopausal status (yes or no, for women only), and other diseases.

## Supplementary materials

Supplementary Material 1. A total of 106 food items in the food frequency questionnaire used in the KoGES

Supplementary Material 2. Example foods of the 412 foods in the food frequency questionnaire by food groups of the National Institute of Agricultural Sciences (NAS)

Supplementary Material 3. Flow chart of the antioxidant database creation

Supplementary Material 4. The coverage of the antioxidants database within each food group

Supplementary Material 5. Major food items contributing to intake and variation of five classes of antioxidants and seven subclasses of flavonoids for participants of general population cohorts in the KoGES

## References

[1] Willett W (2012). Nutritional epidemiology.

[2] Harvard T.H. Chan School of Public Health (2015). Chan School of Public Health. https://www.hsph.harvard.edu/nutrition-questionnaire-service-center/nutrient-tables/.

[3] Kim Y, Han BG, KoGES group (2017). Cohort profile: the Korean Genome and Epidemiology Study (KoGES) consortium. Int J Epidemiol.

[4] Choi E, Ahn S, Joung H (2020). Association of dietary fatty acid consumption patterns with risk of hyper-LDL cholesterolemiain Korean adults. Nutrients.

[5] Kim SY, Woo HW, Lee YH, Shin DH, Shin MH, Choi BY (2020). Association of dietary glycaemic index, glycaemic load, and total carbohydrates with incidence of type-2 diabetes in adults aged ≥ 40 years: the Multi-Rural Communities Cohort (MRCohort). Diabetes Res Clin Pract.

[6] Kim MJ, Woo HW, Shin MH, Koh SB, Kim HC, Kim YM (2024). Habitual intake of iron, copper, and zinc and the risk of type 2 diabetes in a prospective cohort: the CAVAS (Cardiovascular Disease Association Study). Nutr Metab Cardiovasc Dis.

[7] Woo HW, Kim MK, Lee YH, Shin DH, Shin MH, Choi BY (2019). Habitual consumption of soy protein and isoflavones and risk of metabolic syndrome in adults ≥ 40 years old: a prospective analysis of the Korean Multi-Rural Communities Cohort Study (MR-Cohort). Eur J Nutr.

[8] Im J, Park K (2021). Association between soy food and dietary soy isoflavone intake and the risk of cardiovascular disease in women: a prospective cohort study in Korea. Nutrients.

[9] Kim HS, Kwon M, Lee HY, Shivappa N, Hébert JR, Sohn C (2019). Higher pro-inflammatory dietary score is associated with higher hyperuricemia risk: results from the case-controlled Korean Genome and Epidemiology Study_Cardiovascular Disease Association Study. Nutrients.

[10] Sharifi-Rad M, Anil Kumar NV, Zucca P, Varoni EM, Dini L, Panzarini E (2020). Lifestyle, oxidative stress, and antioxidants: back and forth in the pathophysiology of chronic diseases. Front Physiol.

[11] Simioni C, Zauli G, Martelli AM, Vitale M, Sacchetti G, Gonelli A (2018). Oxidative stress: role of physical exercise and antioxidant nutraceuticals in adulthood and aging. Oncotarget.

[12] Joshi P, Kim WJ, Lee SA (2015). The effect of dietary antioxidant on the COPD risk: the community-based KoGES (Ansan-Anseong) cohort. Int J Chron Obstruct Pulmon Dis.

[13] Parohan M, Anjom-Shoae J, Nasiri M, Khodadost M, Khatibi SR, Sadeghi O (2019). Dietary total antioxidant capacity and mortality from all causes, cardiovascular disease and cancer: a systematic review and dose-response meta-analysis of prospective cohort studies. Eur J Nutr.

[14] Pellegrini N, Vitaglione P, Granato D, Fogliano V (2020). Twenty-five years of total antioxidant capacity measurement of foods and biological fluids: merits and limitations. J Sci Food Agric.

[15] Floegel A, Kim DO, Chung SJ, Song WO, Fernandez ML, Bruno RS (2010). Development and validation of an algorithm to establish a total antioxidant capacity database of the US diet. Int J Food Sci Nutr.

[16] Ahn Y, Kwon E, Shim JE, Park MK, Joo Y, Kimm K (2007). Validation and reproducibility of food frequency questionnaire for Korean Genome Epidemiologic Study. Eur J Clin Nutr.

[17] Stadlmayr B, Wijesinha-Bettoni R, Haytowitz D, Rittenschober D, Cunningham J, Sobolewski R (2012). https://www.fao.org/fileadmin/templates/food_composition/documents/upload/INFOODSGuidelinesforFoodMatching_version_1_2.pdf.

[18] National Institute of Agricultural Sciences (2016). Flavonoid data base 1.0. http://koreanfood.rda.go.kr/kfi/fct/fctCompSrch/list.

[19] Del Bo’ C, Bernardi S, Marino M, Porrini M, Tucci M, Guglielmetti S (2019). Systematic review on polyphenol intake and health outcomes: is there sufficient evidence to define a health-promoting polyphenol-rich dietary pattern?. Nutrients.

[20] U.S. Department of Agriculture (USDA) Agricultural Research Service, Nutrient Data Laboratory (2018). SDA database for the flavonoid content of selected foods, release 3.3. https://www.ars.usda.gov/northeast-area/beltsville-md-bhnrc/beltsville-human-nutrition-research-center/methods-and-application-of-food-composition-laboratory/mafcl-site-pages/flavonoids/.

[21] U.S. Department of Agriculture (USDA) Agricultural Research Service, Nutrient Data Laboratory (2015). USDA database for the isoflavone content of selected foods release 2.1. https://www.ars.usda.gov/northeast-area/beltsville-md-bhnrc/beltsville-human-nutrition-research-center/methods-and-application-of-food-composition-laboratory/mafcl-site-pages/isoflavone/.

[22] U.S. Department of Agriculture (USDA) Agricultural Research Service, Nutrient Data Laboratory (2018). USDA database for the proanthocyanidin content of selected foods release 2.1. https://www.ars.usda.gov/northeast-area/beltsville-md-bhnrc/beltsville-human-nutrition-research-center/methods-and-application-of-food-composition-laboratory/mafcl-site-pages/proanthocyanidin/.

[23] Rothwell JA, Perez-Jimenez J, Neveu V, Medina-Remón A, M’hiri N, García-Lobato P (2013). Phenol-Explorer 3.0: a major update of the Phenol-Explorer database to incorporate data on the effects of food processing on polyphenol content. Database (Oxford).

[24] Kim S, Thiessen PA, Bolton EE, Chen J, Fu G, Gindulyte A (2016). PubChem substance and compound databases. Nucleic Acids Res.

[25] Ministry of Food and Drug Safety (2020). Food and nutrient databases. https://various.foodsafetykorea.go.kr/nutrient/.

[26] U.S. Department of Agriculture (USDA) Agricultural Research Service, Nutrient Data Laboratory USDA national nutrient database for standard reference, release 28 (slightly revised) 2016. https://www.ars.usda.gov/northeast-area/beltsville-md-bhnrc/beltsville-human-nutrition-research-center/methods-and-application-of-food-composition-laboratory/mafcl-site-pages/sr11-sr28/.

[27] Watanabe T, Kawai R (2018). Advances in food composition tables in Japan-Standard Tables of Food Composition in Japan - 2015 - (seventh revised edition). Food Chem.

[28] Editorial Board of Encyclopedia of Kimchi (2004). Encyclopedia of kimchi.

[29] U.S. Department of Agriculture (USDA) Agricultural Research Service, Nutrient Data Laboratory USDA’s expanded flavonoid database for the assessment of dietary intakes release 1.1; 2014. https://www.ars.usda.gov/northeast-area/beltsville-md-bhnrc/beltsville-human-nutrition-research-center/methods-and-application-of-food-composition-laboratory/mafcl-site-pages/flavonoids/.

[30] Chun OK, Chung SJ, Song WO (2007). Estimated dietary flavonoid intake and major food sources of U.S. adults. J Nutr.

[31] Kim DO, Lee CY (2004). Comprehensive study on vitamin C equivalent antioxidant capacity (VCEAC) of various polyphenolics in scavenging a free radical and its structural relationship. Crit Rev Food Sci Nutr.

[32] Jun S, Chun OK, Joung H (2018). Estimation of dietary total antioxidant capacity of Korean adults. Eur J Nutr.

[33] Kim K, Vance TM, Chen MH, Chun OK (2018). Dietary total antioxidant capacity is inversely associated with all-cause and cardiovascular disease death of US adults. Eur J Nutr.

[34] Korean Nutrition Society (2011). Nutritional assessment program ‘CANPro 4.0’ [CD-ROM].

[35] Jun S, Shin S, Joung H (2016). Estimation of dietary flavonoid intake and major food sources of Korean adults. Br J Nutr.

[36] Yang YK, Kim JY, Kwon O (2012). Development of flavonoid database for commonly consumed foods by Koreans. Korean J Nutr.

[37] Vogiatzoglou A, Heuer T, Mulligan AA, Lentjes MA, Luben RN, Kuhnle GG (2014). Estimated dietary intakes and sources of flavanols in the German population (German National Nutrition Survey II). Eur J Nutr.

[38] Knaze V, Rothwell JA, Zamora-Ros R, Moskal A, Kyrø C, Jakszyn P (2018). A new food-composition database for 437 polyphenols in 19,899 raw and prepared foods used to estimate polyphenol intakes in adults from 10 European countries. Am J Clin Nutr.

[39] Watanabe S, Zhuo XG, Kimira M (2004). Food safety and epidemiology: new database of functional food factors. Biofactors.

[40] Rural Development Administration (RDA) (2009). Tables of food functional composition.

[41] Kim D, Han A, Park Y (2021). Association of dietary total antioxidant capacity with bone mass and osteoporosis risk in Korean women: analysis of the Korea National Health and Nutrition Examination Survey 2008-2011. Nutrients.

[42] Kim SA, Jun S, Joung H (2016). Estimated dietary intake of vitamin A in Korean adults: based on the Korea National Health and Nutrition Examination Survey 2007~ 2012. J Nutr Health.

[43] Kweon S, Park JY, Park M, Kim Y, Yeon SY, Yoon L (2021). Trends in food and nutrient intake over 20 years: findings from the 1998-2018 Korea National Health and Nutrition Examination Survey. Epidemiol Health.

[44] Kong JS, Kim YM, Woo HW, Shin MH, Koh SB, Kim HC (2023). Prospective associations between cumulative average intake of flavonoids and hypertension risk in the CArdioVascular Disease Association Study (CAVAS). Nutrients.

[45] Kong JS, Lee J, Kim Y, Woo HW, Shin MH, Koh SB (2023). Associations of cumulative average dietary total antioxidant capacity and intake of antioxidants with metabolic syndrome risk in Korean adults aged 40 years and older: a prospective cohort study (KoGES_CAVAS). Epidemiol Health.

[46] Kwon YS, Yang YY, Park Y, Park YK, Kim S (2020). Dietary assessment and factors according to fruits and vegetables intake in Korean elderly people: analysis of data from the Korea National Health and Nutrition Examination Survey, 2013-2018. Nutrients.

[47] Bellavia A, Larsson SC, Bottai M, Wolk A, Orsini N (2013). Fruit and vegetable consumption and all-cause mortality: a dose-response analysis. Am J Clin Nutr.

[48] van der Avoort CM, Ten Haaf DS, de Vries JH, Verdijk LB, van Loon LJ, Eijsvogels TM (2021). Higher levels of physical activity are associated with greater fruit and vegetable intake in older adults. J Nutr Health Aging.

[49] Woo HW, Kim MK, Kong JS, Lee J, Shin MH, Koh SB (2024). The association of dietary total flavonoids and their subclasses with the risk of type 2 diabetes: a prospective cohort study. Eur J Nutr.

