# Supplementary material for: Expansion of a food composition database for the food frequency questionnaire in the Korean Genome and Epidemiology Study (KoGES): a comprehensive database of dietary antioxidants and total antioxidant capacity
Source: Epidemiol Health. 2024 May 10;46:e2024050. doi: 10.4178/epih.e2024050 (PMC11417454; doi:10.4178/epih.e2024050)
Supplement: Supplementary Material 1. — A total of 106 food items in the food frequency questionnaire used in the KoGES [file epih-46-e2024050-Supplementary-1.docx]

**Supplementary Material 1. A total of 106 food items in the food frequency questionnaire used in the KoGES**

| Food group^1^ | Subgroup | Number of food items | Food items |
| --- | --- | --- | --- |
| Cereals | Rice | 5 | Cooked white rice; Cooked rice with beans; Cooked rice with multi-grains; Cooked white rice only or with beans; Cooked rice with beans or with multi-grains |
|  | Noodles | 5 | *Ramyon*; *Kalguksu*/*Jangguk*-noodles/Udon; *Chajangmyon*/*Champpong*; *Naengmyeon*/Buckwheat noodles; Dumpling/Dumpling soup |
|  | Rice Cakes | 2 | Plain stick shaped-rice cake/*Tteokguk* (Plain stick shaped-rice cake soup); Other rice cakes (Steamed rice cake, *Baekseolgi*, *Injeolmi*, etc.) |
|  | Cornflake | 1 | Cornflake |
|  | Grain Powder | 1 | Powdered meals/Parched cereal powder |
|  | Breads | 3 | Loaf bread/Sandwich/Toast; Bread with small red bean; Other breads (Streusel bread/Nut cake/Castella/Cream bread) |
|  | Dessert | 3 | Cake/*Chocopie*; Cookie/Cracker/Snack; Candy/Chocolate |
| Potatoes and Starches | Potatoes and Starch | 4 | Potatoes (Steamed potatoes, French fries, Potato soup, Potato stew, Potato jeon, etc.); Sweet potatoes (Steamed sweet potatoes, *Mattang*, etc.); *Japchae* (Starch vermicelli); *Muk* (Starch jelly) |
| Sugars and Sweeteners | Coffee Additives | 2 | Coffee sugar; Coffee cream |
| Pulses | Legumes | 3 | Soybeans/Soybeans cooked in soy sauce (include green beans, exclude rice with beans); Tofu (Soft tofu, Tofu stew, and Tofu in tofu stew); Soybean paste soup/Fast-fermented bean paste/Soybean paste/*Ssamjang*; |
|  | Soy Milk | 1 | Soy milk |
| Nuts and Seeds | Nuts | 1 | Peanut/Almond/Pine nut |
| Vegetables | Pickled Vegetables, Kimchi, Green Leaf | 2 | *Baechukimchi*/*Baegkimchi*/*Baechukimchi* in Kimchi stew; Other Kimchi (*Pakimchi*/*Kodulbbagi*/*Gatkimchi*) |
|  | Pickled Vegetables, Kimchi, White Root | 2 | *Kkakdugi*/Radish Kimchi; *Nabakkimchi*/*Dongchimi*; |
|  | Pickled vegetables, Other Pickles (*Jang-ajji*) | 1 | Other pickled vegetables (Garlic pickles, Garlic stems, Radish pickles) |
|  | Green Vegetables | 11 | Cabbage/Cabbage soup; Spinach (Spinach namul, Soup, etc.); Lettuce (*Ssam* [rice and condiments wrapped in leaves of lettuce]/Salad, etc.); Perilla leaf; Vegetable wraps/Vegetable salad (Cabbage, Lettuce, Kale, Chicory, Bok choy, Broccoli, etc.); Other green vegetables (Shepherd's purse, Beetroot, Curled mallow, Mugwort, Outer leaves, etc.); Bracken/Sweet potato stems/Taro stem; Red pepper leaves/*Chamnamul*/ *Chwinamul*; Crown daisies/Leek/Water dropwort; Cucumber; Green pepper |
|  | Dark yellow Vegetables | 2 | Carrot/Carrot juice; Pumpkin/Sweet pumpkin/Pumpkin juice |
|  | Other Vegetables | 5 | Bean sprouts/Mung bean sprouts; Radish (Soup, Stew)/ Pickled radish; *Doraji*/*Deoduck* (kind of white root); Onion; Pumpkin, immature |
|  | Tomatoes | 1 | Tomato/Tomato juice/Cherry tomato |
| Mushrooms | Mushrooms | 2 | Oyster mushroom (*Pleurotus ostreatu*s); Other mushrooms (Wood ear mushroom [*Auricularia heimuer*], Button mushroom [*Agaricus bisporus*], Winter mushroom [*Flammulina velutipes*] etc.) |
| Fruits | Fruit | 11 | Strawberries; Korean melon/Melon; Watermelon; Peach/Plum; Banana; Persimmon, hard/Persimmon, dried; Tangerine; Korean pear/Pear juice; Apple/Apple juice; Orange/Orange juice; Grapes/Grapes juice |
|  | Spreads | 1 | Jam/Honey/Margarine |
| Meats | Poultry | 1 | Fried chicken/Whole chicken soup/*Samgyetang*/Chicken stew |
|  | Unprocessed Red Meat | 7 | Pork belly; Pork, Pan roasted/Fried/Pork *bulgogi*/Meatball; Pork, steamed (Boiled pork, Pork braised in soy sauce, Pigs' feet); Steak/beef roast (Grilled ribs, Sirloin, Tenderloin, Beef *bulgogi*); Dog meat; Tang (*Seolleongtang*/*Gomtang*/*Galbitang*/*Doganitang*); Soup (Beef soup, *Yukgaejang*, etc.) |
|  | Processed Red Meat | 2 | Processed meat (Ham, Sausage); By-products (Organ meat, *Seonji*, *Sundae*) |
| Eggs | Eggs | 1 | Egg/Quail egg |
| Fishes | Seafood | 3 | Cuttlefish/Dried cuttlefish/Small octopus; Crab/Crab preserved in soy sauce; Shrimp |
|  | Salted Seafood | 1 | Salt-fermented fish (Salted squid, Salted intestine, Salted pollack roe, Salted shrimp, Salted anchovies, Salted clams, etc.) |
|  | Processed Seafood | 2 | Tuna, canned; Fish paste/Crab, flavored |
|  | Fish | 7 | Sliced raw fish; Blue-colored back fish (Mackerel/Pacific saury/Spanish mackerel); Hair tail; Eel; Yellow croaker/Snapper/Halibut; Alaska pollack/Frozen Alaska pollack/Dried Alaska pollack; Dried anchovy/Stir-fried dried anchovies |
|  | Shellfish | 2 | Clam (Small ark shell/Little neck clam/Clam meat)/Whelk (Soup, Stew, Roast, *Kalguksu*, Salad, etc.); Oysters (Salted oysters) |
| Seaweeds | Seaweeds | 2 | Laver, dried; Kelp/Sea mustard |
| Milks and Milk Products | Dairy Products | 4 | Milk; Yogurt/Yoplait; Ice cream; Cheese; |
| Oils and Fats | - | 0 | - |
| Teas | Other Beverages | 1 | Other beverages (Citron tea, Plum tea, Aloe, Persimmon punch, Ginseng tea, *Sikhye*, Jujube tea, Black herbal tea, etc.) |
|  | Tea | 1 | Green tea |
|  | Coffee | 1 | Coffee |
| Beverages | Carbonated Beverages | 1 | Carbonated drinks (Coke, Sprite) |
| Alcohols | - | 0 | - |
| Seasonings | - | 0 | - |
| Prepared Foods | Pizza/Hamburger | 1 | Pizza/Hamburger |
| Others | - | 0 | - |

^1^ Twenty food groups were based on food categories of the Korean Food Composition Table (ver 9.2) published by the National Institute of Agricultural Sciences (2020)
