# Supplementary material for: Expansion of a food composition database for the food frequency questionnaire in the Korean Genome and Epidemiology Study (KoGES): a comprehensive database of dietary antioxidants and total antioxidant capacity
Source: Epidemiol Health. 2024 May 10;46:e2024050. doi: 10.4178/epih.e2024050 (PMC11417454; doi:10.4178/epih.e2024050)
Supplement: Supplementary Material 2. — Example foods of the 412 foods in the food frequency questionnaire by food groups of the National Institute of Agricultural Sciences (NAS) [file epih-46-e2024050-Supplementary-2.docx]

**Supplementary Material 2. Example foods of the 412 foods in the food frequency questionnaire by food groups of the National Institute of Agricultural Sciences (NAS)**

| Food groups^1^ | Number of foods | Examples of food |
| --- | --- | --- |
| Cereals | 71 | Prosomillet, Polished grain; Buckwheat starch jelly; Wheat flour, hard (strong) flour; Barley, naked barley; etc. |
| Potatoes and Starches | 4 | Potatoes, raw; Sweet potatoes, raw; Starches, potato, powder; Starches, cellophane noodle; etc. |
| Sugars and Sweeteners | 7 | Honey; Sugar, White sugar; Candy, hard; Caramel; etc. |
| Pulses | 11 | Soybean (*Glycine max (L.) Merr.*); Black beans, *Heuktae*; *Kongjorim* (Simmered beans in soy sauce and sugar); Tofu soybean milk, drink (*Vegemeal*); etc. |
| Nuts and Seeds | 6 | Peanut (*Arachis hypogaea*), dried; Almonds, seasoned; Pine nuts, roasted; Sesame, black, roasted; etc. |
| Vegetables | 82 | Chinese cabbage, raw; Broccoli, raw; Onion, raw, domestic; Soybean sprouts, boiled; etc. |
| Mushrooms | 7 | Oyster mushroom, raw; Wood ear (*Auricularia auricula-judae*), blanched; Pine mushroom (*Tricholoma matsutake Sing*.), raw; Cultivated mushroom (*Agaricus bisporus (Lge.) Sing.*), raw; etc. |
| Fruits | 33 | Strawberries, raw, improved; Peach, raw, yellow; Apple, raw, *Hongok*; Orange; etc. |
| Meats | 63 | Beef, Korean cattle, loin; Beef, Korean cattle, *Bulgogi*, Sliced beef with sauce; Pork, ribs, raw; Chicken, meat and skin, raw; etc. |
| Eggs | 2 | Egg, whole, raw; Quail's egg, fresh |
| Fishes | 72 | Anchovy, raw; Alaska pollock, raw; Shrimp, jumbo shrimp, raw; Squid, raw; etc. |
| Seaweeds | 7 | Laver, dried; Seaweed, kelp, dried; Sea mustard, raw, cultured; Sea mustard, stem, raw; etc. |
| Milks and Milk Products | 19 | Cow's milk; Ice cream, vanilla; Yogurt, curd, type; Cheese, mozzarella; etc. |
| Oils and Fats | 5 | Butter; Margarine; Sesame oil; Soybean oil; etc. |
| Teas | 5 | Coffee, powder, instant; Green tea, leaves, dried, infusion; etc. |
| Beverages | 8 | Carbonated beverages, Cola; Carbonated beverages, Cider; etc. |
| Alcohols | 0 | - |
| Seasonings | 6 | Red pepper powder; *Doenjang*; *Ssamjang* (Mixed soybean paste with red pepper paste); *Jajang*, Black bean paste; etc. |
| Prepared Foods | 2 | Pizza; Hamburger, regular |
| Others | 2 | Pine leaves; Leavening agent, yeast, dried |

^1^ Twenty Food groups of the National Institute of Agricultural Sciences, ver. 9.2
