# Supplementary material for: Expansion of a food composition database for the food frequency questionnaire in the Korean Genome and Epidemiology Study (KoGES): a comprehensive database of dietary antioxidants and total antioxidant capacity
Source: Epidemiol Health. 2024 May 10;46:e2024050. doi: 10.4178/epih.e2024050 (PMC11417454; doi:10.4178/epih.e2024050)
Supplement: Supplementary Material 3. — Flow chart of the antioxidant database creation [file epih-46-e2024050-Supplementary-3.docx]

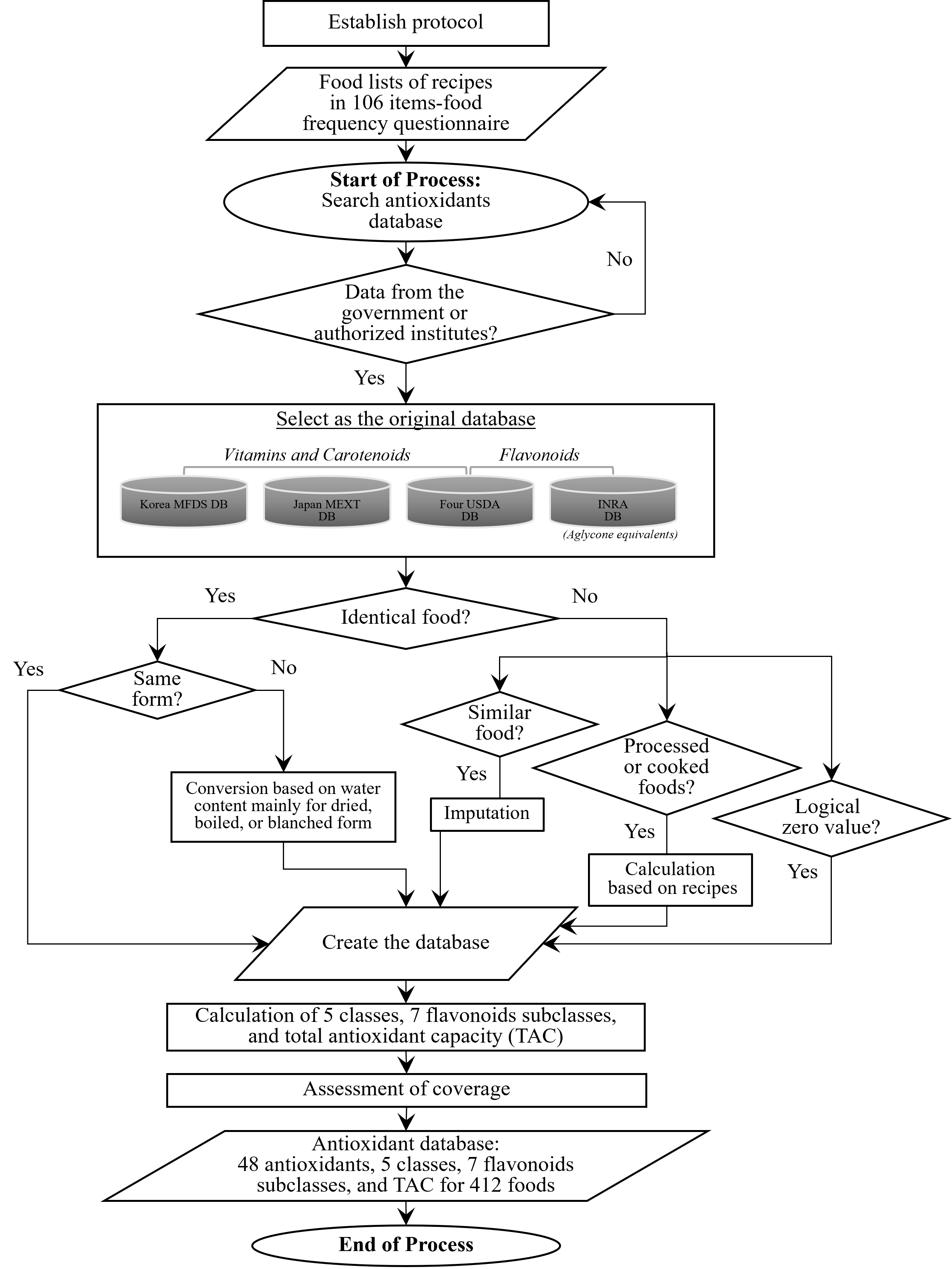


**Supplementary Material 3. Flow chart of the antioxidant database creation**

Abbreviations and accessed date of the original databases:
Korea MFDS, Food and Nutrient Database of the Korea Ministry of Food and Drug Safety (accessed on 17 July 2020); Japan MEXT DB, Standard Tables of Food Composition in Japan (2015) (Seventh Edition) of Ministry of Education, Culture, Sports, Science and Technology(MEXT) Japan (accessed on 6 May 2020); Four USDA DB (1) USDA National Nutrient Database for Standard Reference, Release 28 (2015), (2) USDA Database for the Flavonoid Content of Selected Foods Release 3.3 (2018), (3) USDA Database for the Isoflavone Content of Selected Foods Release 2.1, (4) USDA Database for the Proanthocyanidin Content of Selected Foods Release 2.1 (accessed on 25 May 2020);INRA DB, Phenol-Explorer 3.6 of Institut National de la Recherche Agronomique (accessed on 25 May 2020)
