# Supplementary material for: Expansion of a food composition database for the food frequency questionnaire in the Korean Genome and Epidemiology Study (KoGES): a comprehensive database of dietary antioxidants and total antioxidant capacity
Source: Epidemiol Health. 2024 May 10;46:e2024050. doi: 10.4178/epih.e2024050 (PMC11417454; doi:10.4178/epih.e2024050)
Supplement: Supplementary Material 4. — The coverage of the antioxidants database within each food group [file epih-46-e2024050-Supplementary-4.docx]

**Supplementary** **Material 4. The coverage of the antioxidants database within each food group**

|  | Cereals | | Potatoes and Starches | | Sugars and Sweeteners | | Pulses | | Nuts and Seeds | | Vegetables | | Mushrooms | | Fruits | | Meats | |
| --- | --- | --- | --- | --- | --- | --- | --- | --- | --- | --- | --- | --- | --- | --- | --- | --- | --- | --- |
|  | No. of food | Coverage (%) | No. of food | Coverage (%) | No. of food | Coverage (%) | No. of food | Coverage (%) | No. of food | Coverage (%) | No. of food | Coverage (%) | No. of food | Coverage (%) | No. of food | Coverage (%) | No. of food | Coverage (%) |
| N | 71 |  | 4 |  | 7 |  | 11 |  | 6 |  | 82 |  | 7 |  | 33 |  | 63 |  |
|  |  |  |  |  |  |  |  |  |  |  |  |  |  |  |  |  |  |  |
| **Retinol (μg)**^1^ | 70 | 98.6 | 4 | 100 | 7 | 100 | 11 | 100 | 6 | 100 | 82 | 100 | 7 | 100 | 33 | 100 | 62 | 98.4 |
| **Carotenoids (mg)** |  |  |  |  |  |  |  |  |  |  |  |  |  |  |  |  |  |  |
| Alpha-Carotene (μg) | 53 | 74.6 | 4 | 100 | 7 | 100 | 11 | 100 | 5 | 83.3 | 70 | 85.4 | 7 | 100 | 33 | 100 | 62 | 98.4 |
| Beta-Carotene (μg) | 71 | 100 | 4 | 100 | 7 | 100 | 11 | 100 | 6 | 100 | 82 | 100 | 7 | 100 | 33 | 100 | 63 | 100 |
| Lycopene (μg) | 41 | 57.7 | 4 | 100 | 7 | 100 | 11 | 100 | 5 | 83.3 | 57 | 69.5 | 7 | 100 | 29 | 87.9 | 62 | 98.4 |
| Beta cryptoxanthin (μg) | 53 | 74.6 | 4 | 100 | 7 | 100 | 11 | 100 | 5 | 83.3 | 70 | 85.4 | 7 | 100 | 33 | 100 | 62 | 98.4 |
| Lutein & Zeaxanthin (μg) | 41 | 57.7 | 4 | 100 | 7 | 100 | 11 | 100 | 5 | 83.3 | 57 | 69.5 | 7 | 100 | 29 | 87.9 | 62 | 98.4 |
| **Vitamin C (mg)** | 70 | 98.6 | 4 | 100 | 7 | 100 | 11 | 100 | 6 | 100 | 82 | 100 | 7 | 100 | 33 | 100 | 62 | 98.4 |
| **Vitamin E (mg)** |  |  |  |  |  |  |  |  |  |  |  |  |  |  |  |  |  |  |
| Alpha-tocopherol (mg) | 70 | 98.6 | 4 | 100 | 7 | 100 | 11 | 100 | 6 | 100 | 82 | 100 | 7 | 100 | 33 | 100 | 62 | 98.4 |
| Beta-tocopherol (mg) | 69 | 97.2 | 4 | 100 | 7 | 100 | 11 | 100 | 6 | 100 | 82 | 100 | 7 | 100 | 33 | 100 | 62 | 98.4 |
| Gamma-tocopherol (mg) | 69 | 97.2 | 4 | 100 | 7 | 100 | 11 | 100 | 6 | 100 | 82 | 100 | 7 | 100 | 33 | 100 | 62 | 98.4 |
| Delta-tocopherol (mg) | 69 | 97.2 | 4 | 100 | 7 | 100 | 11 | 100 | 6 | 100 | 82 | 100 | 7 | 100 | 33 | 100 | 62 | 98.4 |
| **Total flavonoids (mg)** |  |  |  |  |  |  |  |  |  |  |  |  |  |  |  |  |  |  |
| **Flavonols (mg)** |  |  |  |  |  |  |  |  |  |  |  |  |  |  |  |  |  |  |
| Quercetin (mg) | 0 | 0 | 2 | 50 | 1 | 14.3 | 5 | 45.5 | 4 | 66.7 | 56 | 68.3 | 6 | 85.7 | 31 | 93.9 | 63 | 100 |
| Kaempferol (mg) | 0 | 0 | 2 | 50 | 1 | 14.3 | 5 | 45.5 | 2 | 33.3 | 56 | 68.3 | 6 | 85.7 | 29 | 87.9 | 63 | 100 |
| Myricetin (mg) | 0 | 0 | 2 | 50 | 1 | 14.3 | 5 | 45.5 | 4 | 66.7 | 48 | 58.5 | 6 | 85.7 | 27 | 81.8 | 63 | 100 |
| Isorhamnetin (mg) | 0 | 0 | 0 | 0 | 1 | 14.3 | 0 | 0 | 1 | 16.7 | 20 | 24.4 | 0 | 0 | 9 | 27.3 | 63 | 100 |
| **Flavones (mg)** |  |  |  |  |  |  |  |  |  |  |  |  |  |  |  |  |  |  |
| Luteolin (mg) | 2 | 2.8 | 2 | 50 | 1 | 14.3 | 5 | 45.5 | 2 | 33.3 | 43 | 52.4 | 6 | 85.7 | 27 | 81.8 | 63 | 100 |
| Apigenin (mg) | 3 | 4.2 | 2 | 50 | 1 | 14.3 | 1 | 9.1 | 4 | 66.7 | 38 | 46.3 | 6 | 85.7 | 25 | 75.8 | 63 | 100 |
| **Flavanones (mg)** |  |  |  |  |  |  |  |  |  |  |  |  |  |  |  |  |  |  |
| Hesperetin (mg) | 0 | 0 | 1 | 25 | 0 | 0 | 0 | 0 | 4 | 66.7 | 10 | 12.2 | 0 | 0 | 20 | 60.6 | 63 | 100 |
| Naringenin (mg) | 2 | 2.8 | 1 | 25 | 0 | 0 | 0 | 0 | 4 | 66.7 | 6 | 7.3 | 0 | 0 | 18 | 54.5 | 63 | 100 |
| Eriodictyol (mg) | 0 | 0 | 0 | 0 | 0 | 0 | 0 | 0 | 1 | 16.7 | 2 | 2.4 | 0 | 0 | 5 | 15.2 | 63 | 100 |
| Flavan-3-ols (mg) |  |  |  |  |  |  |  |  |  |  |  |  |  |  |  |  |  |  |
| Catechin (mg) | 7 | 9.9 | 1 | 25 | 1 | 14.3 | 1 | 9.1 | 4 | 66.7 | 16 | 19.5 | 6 | 85.7 | 27 | 81.8 | 63 | 100 |
| Epicatechin (mg) | 9 | 12.7 | 1 | 25 | 1 | 14.3 | 4 | 36.4 | 4 | 66.7 | 15 | 18.3 | 6 | 85.7 | 27 | 81.8 | 63 | 100 |
| Epigallocatechin (mg) | 5 | 7 | 1 | 25 | 1 | 14.3 | 1 | 9.1 | 4 | 66.7 | 15 | 18.3 | 6 | 85.7 | 27 | 81.8 | 63 | 100 |
| Epicatechin 3-gallate (mg) | 5 | 7 | 1 | 25 | 1 | 14.3 | 1 | 9.1 | 4 | 66.7 | 15 | 18.3 | 6 | 85.7 | 27 | 81.8 | 63 | 100 |
| Epigallocatechin 3-gallate (mg) | 5 | 7 | 1 | 25 | 1 | 14.3 | 1 | 9.1 | 4 | 66.7 | 15 | 18.3 | 6 | 85.7 | 27 | 81.8 | 63 | 100 |
| Gallocatechin (mg) ^1^ | 5 | 7 | 1 | 25 | 1 | 14.3 | 1 | 9.1 | 4 | 66.7 | 15 | 18.3 | 6 | 85.7 | 27 | 81.8 | 63 | 100 |
| Theaflavin (mg) | 71 | 100 | 4 | 100 | 7 | 100 | 1 | 9.1 | 6 | 100 | 82 | 100 | 7 | 100 | 33 | 100 | 63 | 100 |
| Thearubigin (mg) | 71 | 100 | 4 | 100 | 7 | 100 | 11 | 100 | 6 | 100 | 82 | 100 | 7 | 100 | 33 | 100 | 63 | 100 |
| Theaflavin 3-gallate (mg) | 71 | 100 | 4 | 100 | 7 | 100 | 11 | 100 | 6 | 100 | 82 | 100 | 7 | 100 | 33 | 100 | 63 | 100 |
| Theaflavin 3'-gallate (mg) | 71 | 100 | 4 | 100 | 7 | 100 | 11 | 100 | 6 | 100 | 82 | 100 | 7 | 100 | 33 | 100 | 63 | 100 |
| Theaflavin 3,3'-digallate (mg) | 71 | 100 | 4 | 100 | 7 | 100 | 11 | 100 | 6 | 100 | 82 | 100 | 7 | 100 | 33 | 100 | 63 | 100 |
| **Anthocyanins (mg)** |  |  |  |  |  |  |  |  |  |  |  |  |  |  |  |  |  |  |
| Cyanidin (mg) | 54 | 76.1 | 3 | 75 | 6 | 85.7 | 5 | 45.5 | 4 | 66.7 | 27 | 32.9 | 4 | 57.1 | 20 | 60.6 | 63 | 100 |
| Delphinidin (mg) | 54 | 76.1 | 3 | 75 | 6 | 85.7 | 6 | 54.5 | 6 | 100 | 24 | 29.3 | 4 | 57.1 | 17 | 51.5 | 63 | 100 |
| Malvidin (mg) | 54 | 76.1 | 3 | 75 | 6 | 85.7 | 6 | 54.5 | 6 | 100 | 23 | 28 | 4 | 57.1 | 17 | 51.5 | 63 | 100 |
| Pelargonidin (mg) | 54 | 76.1 | 3 | 75 | 6 | 85.7 | 5 | 45.5 | 6 | 100 | 24 | 29.3 | 4 | 57.1 | 16 | 48.5 | 63 | 100 |
| Peonidin (mg) | 54 | 76.1 | 3 | 75 | 6 | 85.7 | 5 | 45.5 | 6 | 100 | 23 | 28 | 4 | 57.1 | 17 | 51.5 | 63 | 100 |
| Petunidin (mg) | 54 | 76.1 | 3 | 75 | 6 | 85.7 | 6 | 54.5 | 6 | 100 | 23 | 28 | 4 | 57.1 | 16 | 48.5 | 63 | 100 |
| **Isoflavones (mg)** |  |  |  |  |  |  |  |  |  |  |  |  |  |  |  |  |  |  |
| Daidzein (mg) | 33 | 46.5 | 2 | 50 | 0 | 0 | 11 | 100 | 4 | 66.7 | 41 | 50 | 6 | 85.7 | 25 | 75.8 | 63 | 100 |
| Genistein (mg) | 33 | 46.5 | 2 | 50 | 0 | 0 | 11 | 100 | 4 | 66.7 | 41 | 50 | 6 | 85.7 | 25 | 75.8 | 63 | 100 |
| Glycitein (mg) | 20 | 28.2 | 2 | 50 | 0 | 0 | 11 | 100 | 3 | 50 | 11 | 13.4 | 0 | 0 | 7 | 21.2 | 63 | 100 |
| Biochanin (mg) | 11 | 15.5 | 0 | 0 | 0 | 0 | 9 | 81.8 | 1 | 16.7 | 5 | 6.1 | 0 | 0 | 4 | 12.1 | 63 | 100 |
| Formononetin (mg) ^1^ |  |  | 1 | 25 | 0 | 0 | 11 | 100 | 4 | 66.7 | 11 | 13.4 | 0 | 0 | 7 | 21.2 | 63 | 100 |
| **Proanthocyanidins (mg)** | 15 | 21.1 |  |  |  |  |  |  |  |  |  |  |  |  |  |  |  |  |
| Dimer (mg) | 27 | 38 | 2 | 50 | 1 | 14.3 | 5 | 45.5 | 4 | 66.7 | 35 | 42.7 | 0 | 0 | 30 | 90.9 | 63 | 100 |
| Trimer (mg) | 27 | 38 | 2 | 50 | 1 | 14.3 | 5 | 45.5 | 4 | 66.7 | 35 | 42.7 | 0 | 0 | 27 | 81.8 | 63 | 100 |
| 4-6 monomers (mg) ^1^ | 25 | 35.2 | 2 | 50 | 1 | 14.3 | 4 | 36.4 | 4 | 66.7 | 32 | 39 | 0 | 0 | 24 | 72.7 | 63 | 100 |
| 7-10 monomers (mg) ^1^ | 25 | 35.2 | 2 | 50 | 1 | 14.3 | 3 | 27.3 | 4 | 66.7 | 32 | 39 | 0 | 0 | 24 | 72.7 | 63 | 100 |
| Polymer (mg) ^1^ | 21 | 29.6 | 2 | 50 | 1 | 14.3 | 3 | 27.3 | 4 | 66.7 | 32 | 39 | 0 | 0 | 24 | 72.7 | 63 | 100 |

^1^ Components (retinol; gallocatechin of flavan-3-olds; formonoetin of isoflavones; 4-6 monomers, 7-10 monomers, and polymers of proanthocyanidins) were not included to estimate dietary total antioxidant capacity.

**Supplementary Material 4. Continued**

|  | Eggs | | Fishes | | Seaweeds | | Milks and Milk Products | | Oils and Fats | | Teas | | Beverages | | Seasonings | | Prepared Foods | | Others | |
| --- | --- | --- | --- | --- | --- | --- | --- | --- | --- | --- | --- | --- | --- | --- | --- | --- | --- | --- | --- | --- |
|  | No. of food | Coverage (%) | No. of food | Coverage (%) | No. of food | Coverage (%) | No. of food | Coverage (%) | No. of food | Coverage (%) | No. of food | Coverage (%) | No. of food | Coverage (%) | No. of food | Coverage (%) | No. of food | Coverage (%) | No. of food | Coverage (%) |
| N | 2 |  | 72 |  | 7 |  | 19 |  | 5 |  | 5 |  | 8 |  | 6 |  | 2 |  | 2 |  |
|  |  |  |  |  |  |  |  |  |  |  |  |  |  |  |  |  |  |  |  |  |
| **Retinol (μg)**^1^ | 2 | 100 | 72 | 100 | 7 | 100 | 18 | 94.7 | 5 | 100 | 5 | 100 | 8 | 100 | 6 | 100 | 2 | 100 | 2 | 100 |
| **Carotenoids (mg)** |  |  |  |  |  |  |  |  |  |  |  |  |  |  |  |  |  |  |  |  |
| Alpha-Carotene (μg) | 2 | 100 | 72 | 100 | 7 | 100 | 19 | 100 | 5 | 100 | 4 | 80 | 8 | 100 | 6 | 100 | 2 | 100 | 1 | 50 |
| Beta-Carotene (μg) | 2 | 100 | 72 | 100 | 7 | 100 | 19 | 100 | 5 | 100 | 5 | 100 | 8 | 100 | 6 | 100 | 2 | 100 | 2 | 100 |
| Lycopene (μg) | 2 | 100 | 72 | 100 | 7 | 100 | 19 | 100 | 5 | 100 | 4 | 80 | 8 | 100 | 6 | 100 | 2 | 100 | 1 | 50 |
| Beta Cryptoxanthin (μg) | 2 | 100 | 72 | 100 | 7 | 100 | 19 | 100 | 5 | 100 | 4 | 80 | 8 | 100 | 6 | 100 | 2 | 100 | 1 | 50 |
| Lutein & Zeaxanthin (μg) | 2 | 100 | 72 | 100 | 7 | 100 | 19 | 100 | 5 | 100 | 4 | 80 | 8 | 100 | 6 | 100 | 2 | 100 | 1 | 50 |
| **Vitamin C (mg)** | 2 | 100 | 72 | 100 | 7 | 100 | 18 | 94.7 | 5 | 100 | 5 | 100 | 8 | 100 | 6 | 100 | 2 | 100 | 2 | 100 |
| **Vitamin E (mg)** |  |  |  |  |  |  |  |  |  |  |  |  |  |  |  |  |  |  |  |  |
| Alpha-tocopherol (mg) | 2 | 100 | 72 | 100 | 7 | 100 | 18 | 94.7 | 5 | 100 | 5 | 100 | 7 | 87.5 | 6 | 100 | 2 | 100 | 2 | 100 |
| Beta-tocopherol (mg) | 2 | 100 | 72 | 100 | 7 | 100 | 16 | 84.2 | 5 | 100 | 5 | 100 | 7 | 87.5 | 6 | 100 | 2 | 100 | 2 | 100 |
| Gamma-tocopherol (mg) | 2 | 100 | 72 | 100 | 7 | 100 | 16 | 84.2 | 5 | 100 | 5 | 100 | 7 | 87.5 | 6 | 100 | 2 | 100 | 2 | 100 |
| Delta-tocopherol (mg) | 2 | 100 | 72 | 100 | 7 | 100 | 16 | 84.2 | 5 | 100 | 5 | 100 | 7 | 87.5 | 6 | 100 | 2 | 100 | 2 | 100 |
| **Total Flavonoids (mg)** |  |  |  |  |  |  |  |  |  |  |  |  |  |  |  |  |  |  |  |  |
| **Flavonols (mg)** |  |  |  |  |  |  |  |  |  |  |  |  |  |  |  |  |  |  |  |  |
| Quercetin (mg) | 2 | 100 | 72 | 100 | 0 | 0 | 19 | 100 | 5 | 100 | 3 | 60 | 0 | 0 | 2 | 33.3 | 1 | 50 | 0 | 0 |
| Kaempferol (mg) | 2 | 100 | 72 | 100 | 0 | 0 | 19 | 100 | 5 | 100 | 3 | 60 | 0 | 0 | 2 | 33.3 | 1 | 50 | 0 | 0 |
| Myricetin (mg) | 2 | 100 | 72 | 100 | 0 | 0 | 19 | 100 | 5 | 100 | 3 | 60 | 0 | 0 | 1 | 16.7 | 1 | 50 | 0 | 0 |
| Isorhamnetin (mg) | 2 | 100 | 72 | 100 | 0 | 0 | 19 | 100 | 5 | 100 | 1 | 20 | 0 | 0 | 0 | 0 | 1 | 50 | 0 | 0 |
| **Flavones (mg)** |  |  |  |  |  |  |  |  |  |  |  |  |  |  |  |  |  |  |  |  |
| Luteolin (mg) | 2 | 100 | 72 | 100 | 0 | 0 | 19 | 100 | 5 | 100 | 3 | 60 | 0 | 0 | 1 | 16.7 | 1 | 50 | 0 | 0 |
| Apigenin (mg) | 2 | 100 | 72 | 100 | 0 | 0 | 19 | 100 | 5 | 100 | 3 | 60 | 0 | 0 | 1 | 16.7 | 1 | 50 | 0 | 0 |
| **Flavanones (mg)** |  |  |  |  |  |  |  |  |  |  |  |  |  |  |  |  |  |  |  |  |
| Hesperetin (mg) | 2 | 100 | 72 | 100 | 0 | 0 | 19 | 100 | 5 | 100 | 2 | 40 | 0 | 0 | 0 | 0 | 1 | 50 | 0 | 0 |
| Naringenin (mg) | 2 | 100 | 72 | 100 | 0 | 0 | 19 | 100 | 5 | 100 | 1 | 20 | 0 | 0 | 0 | 0 | 1 | 50 | 0 | 0 |
| Eriodictyol (mg) | 2 | 100 | 72 | 100 | 0 | 0 | 19 | 100 | 5 | 100 | 1 | 20 | 0 | 0 | 0 | 0 | 1 | 50 | 0 | 0 |
| Flavan-3-ols (mg) |  |  |  |  |  |  |  |  |  |  |  |  |  |  |  |  |  |  |  |  |
| Catechin (mg) | 2 | 100 | 72 | 100 | 0 | 0 | 19 | 100 | 5 | 100 | 3 | 60 | 0 | 0 | 1 | 16.7 | 1 | 50 | 0 | 0 |
| Epicatechin (mg) | 2 | 100 | 72 | 100 | 0 | 0 | 19 | 100 | 5 | 100 | 3 | 60 | 0 | 0 | 1 | 16.7 | 1 | 50 | 0 | 0 |
| Epigallocatechin (mg) | 2 | 100 | 72 | 100 | 0 | 0 | 19 | 100 | 5 | 100 | 3 | 60 | 0 | 0 | 1 | 16.7 | 1 | 50 | 0 | 0 |
| Epicatechin 3-gallate (mg) | 2 | 100 | 72 | 100 | 0 | 0 | 19 | 100 | 5 | 100 | 2 | 40 | 0 | 0 | 1 | 16.7 | 1 | 50 | 0 | 0 |
| Epigallocatechin 3-gallate (mg) | 2 | 100 | 72 | 100 | 0 | 0 | 19 | 100 | 5 | 100 | 3 | 60 | 0 | 0 | 1 | 16.7 | 1 | 50 | 0 | 0 |
| Gallocatechin (mg) ^1^ | 2 | 100 | 72 | 100 | 0 | 0 | 19 | 100 | 5 | 100 | 3 | 60 | 0 | 0 | 1 | 16.7 | 1 | 50 | 0 | 0 |
| Theaflavin (mg) | 2 | 100 | 72 | 100 | 7 | 100 | 19 | 100 | 5 | 100 | 5 | 100 | 8 | 100 | 6 | 100 | 2 | 100 | 2 | 100 |
| Thearubigin (mg) | 2 | 100 | 72 | 100 | 7 | 100 | 19 | 100 | 5 | 100 | 5 | 100 | 8 | 100 | 6 | 100 | 2 | 100 | 2 | 100 |
| Theaflavin 3-gallate (mg) | 2 | 100 | 72 | 100 | 7 | 100 | 19 | 100 | 5 | 100 | 5 | 100 | 8 | 100 | 6 | 100 | 2 | 100 | 2 | 100 |
| Theaflavin 3'-gallate (mg) | 2 | 100 | 72 | 100 | 7 | 100 | 19 | 100 | 5 | 100 | 5 | 100 | 8 | 100 | 6 | 100 | 2 | 100 | 2 | 100 |
| Theaflavin 3,3'-digallate (mg) | 2 | 100 | 72 | 100 | 7 | 100 | 19 | 100 | 5 | 100 | 5 | 100 | 8 | 100 | 6 | 100 | 2 | 100 | 2 | 100 |
| **Anthocyanins (mg)** |  |  |  |  |  |  |  |  |  |  |  |  |  |  |  |  |  |  |  |  |
| Cyanidin (mg) | 2 | 100 | 72 | 100 | 0 | 0 | 19 | 100 | 5 | 100 | 2 | 40 | 7 | 87.5 | 5 | 83.3 | 1 | 50 | 0 | 0 |
| Delphinidin (mg) | 2 | 100 | 72 | 100 | 0 | 0 | 19 | 100 | 5 | 100 | 2 | 40 | 7 | 87.5 | 4 | 66.7 | 1 | 50 | 0 | 0 |
| Malvidin (mg) | 2 | 100 | 72 | 100 | 0 | 0 | 19 | 100 | 5 | 100 | 2 | 40 | 7 | 87.5 | 4 | 66.7 | 1 | 50 | 0 | 0 |
| Pelargonidin (mg) | 2 | 100 | 72 | 100 | 0 | 0 | 19 | 100 | 5 | 100 | 2 | 40 | 7 | 87.5 | 4 | 66.7 | 1 | 50 | 0 | 0 |
| Peonidin (mg) | 2 | 100 | 72 | 100 | 0 | 0 | 19 | 100 | 5 | 100 | 2 | 40 | 7 | 87.5 | 4 | 66.7 | 1 | 50 | 0 | 0 |
| Petunidin (mg) | 2 | 100 | 72 | 100 | 0 | 0 | 19 | 100 | 5 | 100 | 2 | 40 | 7 | 87.5 | 4 | 66.7 | 1 | 50 | 0 | 0 |
| **Isoflavones (mg)** |  |  |  |  |  |  |  |  |  |  |  |  |  |  |  |  |  |  |  |  |
| Daidzein (mg) | 2 | 100 | 72 | 100 | 0 | 0 | 19 | 100 | 5 | 100 | 3 | 60 | 0 | 0 | 5 | 83.3 | 2 | 100 | 0 | 0 |
| Genistein (mg) | 2 | 100 | 72 | 100 | 0 | 0 | 19 | 100 | 5 | 100 | 3 | 60 | 0 | 0 | 5 | 83.3 | 2 | 100 | 0 | 0 |
| Glycitein (mg) | 2 | 100 | 72 | 100 | 0 | 0 | 19 | 100 | 5 | 100 | 3 | 60 | 0 | 0 | 4 | 66.7 | 2 | 100 | 0 | 0 |
| Biochanin (mg) | 2 | 100 | 72 | 100 | 0 | 0 | 19 | 100 | 5 | 100 | 1 | 20 | 0 | 0 | 1 | 16.7 | 1 | 50 | 0 | 0 |
| Formononetin (mg) ^1^ | 2 | 100 | 72 | 100 | 0 | 0 | 19 | 100 | 5 | 100 | 3 | 60 | 0 | 0 | 4 | 66.7 | 1 | 50 | 0 | 0 |
| **Proanthocyanidins (mg)** |  |  |  |  |  |  |  |  |  |  |  |  |  |  |  |  |  |  |  |  |
| Dimer (mg) | 2 | 100 | 72 | 100 | 0 | 0 | 19 | 100 | 5 | 100 | 3 | 60 | 0 | 0 | 0 | 0 | 1 | 50 | 0 | 0 |
| Trimer (mg) | 2 | 100 | 72 | 100 | 0 | 0 | 19 | 100 | 5 | 100 | 3 | 60 | 0 | 0 | 0 | 0 | 1 | 50 | 0 | 0 |
| 4-6 monomers (mg) ^1^ | 2 | 100 | 72 | 100 | 0 | 0 | 19 | 100 | 5 | 100 | 3 | 60 | 0 | 0 | 0 | 0 | 1 | 50 | 0 | 0 |
| 7-10 monomers (mg) ^1^ | 2 | 100 | 72 | 100 | 0 | 0 | 19 | 100 | 5 | 100 | 3 | 60 | 0 | 0 | 0 | 0 | 1 | 50 | 0 | 0 |
| Polymer (mg) ^1^ | 2 | 100 | 72 | 100 | 0 | 0 | 19 | 100 | 5 | 100 | 3 | 60 | 0 | 0 | 0 | 0 | 1 | 50 | 0 | 0 |

^1^ Components (retinol; gallocatechin of flavan-3-olds; formonoetin of isoflavones; 4-6 monomers, 7-10 monomers, and polymers of proanthocyanidins) were not included to estimate dietary total antioxidant capacity
