# Supplementary material for: Expansion of a food composition database for the food frequency questionnaire in the Korean Genome and Epidemiology Study (KoGES): a comprehensive database of dietary antioxidants and total antioxidant capacity
Source: Epidemiol Health. 2024 May 10;46:e2024050. doi: 10.4178/epih.e2024050 (PMC11417454; doi:10.4178/epih.e2024050)
Supplement: Supplementary Material 5. — Major food items contributing to intake and variation of five classes of antioxidants and seven subclasses of flavonoids for participants of general population cohorts in the KoGES [file epih-46-e2024050-Supplementary-5.docx]

**Supplementary Material 5. Major food items contributing to intake and variation of five classes of antioxidants and seven subclasses of flavonoids for participants of general population cohorts in the KoGES**

| Food items of FFQ | Amount per serving (mg/ml) | Total | | Men | | Women | |
| --- | --- | --- | --- | --- | --- | --- | --- |
|  |  | r^2^ | Intake (%) | r^2^ | Intake (%) | r^2^ | Intake (%) |
| **Total antioxidant capacity** |  |  |  |  |  |  |  |
| Green tea | 120 | 89.6 | 25.9 | 92.3 | 28.2 | 88.3 | 24.7 |
| Apple/Apple juice | 200 | - | 7.42 | - | 6.35 | 5.10 | 8.01 |
| Grapes/Grapes juice | 200 | - | 6.60 | - | 5.60 | - | 7.10 |
| Strawberries | 150 | - | 5.35 | - | - | - | 5.61 |
|  |  |  |  |  |  |  |  |
| **Five classes of antioxidants** |  |  |  |  |  |  |  |
| **Retinol** |  |  |  |  |  |  |  |
| Milk | 200 | 62.9 | 32.3 | 59.1 | 26.7 | 64.9 | 35.3 |
| Eel | 50 | 13.5 | 6.08 | 15.1 | 8.03 | 12.6 | 5.02 |
| Egg/Quail egg | 50 | 8.71 | 20.5 | 9.54 | 21.4 | 8.27 | 20.0 |
| Cornflake | 30 | 5.6 | 5.71 | 5.72 | - | 5.60 | - |
| By-products (Organ meat, Seonji, Sundae) | 50 | - | 5.29 | - | 7.91 | - | - |
| Ice cream | 120 | - | - | - | 5.43 | - | 5.22 |
| Pork belly | 150 | - | - | - | 6.03 | - | - |
| **Carotenoids** |  |  |  |  |  |  |  |
| Tomato/Tomato juice/Cherry tomato | 200 | 66.1 | 24.1 | 63.7 | 22.4 | 66.8 | 25.0 |
| Spinach (Spinach namul, Soup, etc.) | 50 | 17.8 | 13.5 | 20.9 | 14.7 | 16.8 | 12.8 |
| Watermelon | 150 | 6.97 | 13.3 | 6.20 | 12.3 | 7.18 | 13.8 |
| Baechukimchi/Baegkimchi/Baechukimchi in Kimchi stew | 50 | - | 5.97 | - | 6.86 | - | 5.48 |
| Pumpkin, immature | 70 | - | 5.03 | - | - | - | 5.40 |
| Persimmon, hard/Persimmon, dried | 150 | - | - | - | - | - | 5.14 |
| **Vitamin** C |  |  |  |  |  |  |  |
| Orange/Orange juice | 200 | 37.0 | 7.17 | 36.9 | 6.77 | 36.9 | 7.39 |
| Strawberries | 150 | 18.8 | 11.8 | 19.4 | 11.1 | 18.3 | 12.1 |
| Tangerine | 80 | 11.0 | 8.23 | 7.61 | 6.89 | 11.9 | 8.95 |
| Other green vegetables (Shepherd's purse, Beetroot, Curled mallow, Mugwort, Outer leaves, etc.) | 50 | 9.86 | - | 10.7 | - | 9.80 | - |
| Baechukimchi/Baegkimchi/Baechukimchi in Kimchi stew | 50 | - | 15.0 | - | 17.4 | - | 13.7 |
| Green pepper | 20 | - | 6.03 | - | 6.11 | - | 5.99 |
| Sweet potatoes (Steamed sweet potatoes, Mattang, etc.) | 75 | - | - | - | - | - | 5.52 |
| Apple/Apple juice | 200 | - | - | - | - | - | 5.28 |
| **Vitamin E** |  |  |  |  |  |  |  |
| Tofu (Soft tofu, Tofu stew, and Tofu in tofu stew) | 60 | 34.8 | 20.1 | 32.0 | 19.3 | 36.4 | 20.6 |
| Soy milk | 200 | 20.5 | 5.25 | 20.6 | 5.38 | 20.5 | 5.18 |
| Soybeans/Soybeans cooked in soy sauce (include green beans, exclude rice with beans) | 12 | 7.90 | - | 6.57 | - | 8.40 | - |
| Chajangmyon/Champpong | 430 | 6.04 | - | 8.00 | 6.26 | - | - |
| Cooked rice with beans | 220 | 6.1 | - | 7.89 | - | 5.42 | - |
| Cooked rice with multi-grains | 220 | - | 5.30 | - | - | - | 5.74 |
| Ramyon | 120 | - | - | - | 6.96 | - | - |
| Dumpling/Dumpling soup | 200 | - | - | - | - | 5.52 | - |
| **Flavonoids** |  |  |  |  |  |  |  |
| Green tea | 120 | 69.5 | 21.4 | 76.6 | 23.7 | 66.7 | 20.1 |
| Apple/Apple juice | 200 | 20.5 | 15.4 | 15.0 | 13.5 | 22.5 | 16.4 |
| Grapes/Grapes juice | 200 | 5.83 | 10.5 | - | 9.23 | 6.36 | 11.2 |
| Strawberries | 150 | - | 7.64 | - | 7.11 | - | 7.92 |
| Cooked rice with multi-grains | 220 | - | 6.25 | - | 5.95 | - | 6.41 |
|  |  |  |  |  |  |  |  |
| **Seven subclasses of flavonoids** |  |  |  |  |  |  |  |
| **Flavonols** |  |  |  |  |  |  |  |
| Lettuce (Ssam (rice and condiments wrapped in leaves of lettuce)/Salad, etc.) | 25 | 53.8 | 14.2 | 49.7 | 13.1 | 55.7 | 14.8 |
| Other green vegetables (Shepherd's purse, Beetroot, Curled mallow, Mugwort, Outer leaves, etc.) | 50 | 14.5 | 15.8 | 10.2 | 15.0 | 15.1 | 16.3 |
| Green tea | 120 | 12.8 | 10.1 | 15.9 | 10.9 | 11.9 | 9.73 |
| Other Kimchi (Pakimchi/Kodulbbagi/Gatkimchi) | 50 | 9.5 | 5.43 | 14.4 | 6.24 | 8.19 | 4.99 |
| Kkakdugi/Radish Kimchi | 50 | - | 7.69 | - | 9.17 | - | 6.88 |
| Onion | 10 | - | 7.37 | - | 6.34 | - | 7.94 |
| Baechukimchi/Baegkimchi/Baechukimchi in Kimchi stew | 50 | - | 5.69 | - | 6.01 | - | 5.52 |
| Green pepper | 20 | - | 5.68 | - | 5.22 | - | 5.93 |
| Coffee | 2.7 | - | - | - | 5.17 | - | - |
| **Flavones** |  |  |  |  |  |  |  |
| Orange/Orange juice | 200 | 34.8 | 10.5 | 33.7 | 9.56 | 35.0 | 11.0 |
| Green pepper | 20 | 28.4 | 18.1 | 28.2 | 17.5 | 28.4 | 18.3 |
| Tangerine | 80 | 13.2 | 13.0 | 8.68 | 10.6 | 14.7 | 14.3 |
| Green tea | 120 | 7.17 | 7.20 | 10.2 | 8.10 | 6.43 | 6.70 |
| Baechukimchi/Baegkimchi/Baechukimchi in Kimchi stew | 50 | - | 13.4 | - | 14.9 | - | 12.5 |
| Kkakdugi/Radish Kimchi | 50 | - | 6.26 | 6.73 | 7.77 | - | 5.44 |
| Lettuce (Ssam (rice and condiments wrapped in leaves of lettuce)/Salad, etc.) | 25 | - | 5.92 | - | 5.86 | - | 5.95 |
| Watermelon | 150 | - | - | - | - | - | 5.09 |
| **Flavanones** |  |  |  |  |  |  |  |
| Orange/Orange juice | 200 | 67.4 | 30.0 | 74.4 | 30.7 | 65.4 | 29.6 |
| Tangerine | 80 | 25.4 | 39.6 | 19.0 | 38.0 | 27.2 | 40.5 |
| Grapes/Grapes juice | 200 | 7.14 | 25.2 | 6.58 | 25.0 | 7.41 | 25.4 |
| **Flavan-3-ols** |  |  |  |  |  |  |  |
| Green tea | 120 | 99.6 | 47.1 | 99.7 | 49.2 | 99.6 | 45.9 |
| Grapes/Grapes juice | 200 | - | 11.9 | - | 10.4 | - | 12.7 |
| Apple/Apple juice | 200 | - | 10.9 | - | 9.58 | - | 11.6 |
| Coffee | 2.7 | - | - | - | 6.63 | - | - |
| **Anthocyanins** |  |  |  |  |  |  |  |
| Grapes/Grapes juice | 200 | 86.9 | 43.9 | 85.6 | 42.2 | 87.1 | 44.8 |
| Strawberries | 150 | 10.6 | 29.5 | 11.6 | 30.2 | 10.3 | 29.1 |
| Apple/Apple juice | 200 | - | 10.6 | - | 10.3 | - | 10.8 |
| Vegetable wraps/Vegetable salad (Cabbage, Lettuce, Kale, Chicory, Bok choy, Broccoli, etc.) | 50 | - | 6.86 | - | 7.19 | - | 6.68 |
| **Isoflavones** |  |  |  |  |  |  |  |
| Soybeans/Soybeans cooked in soy sauce (include green beans, exclude rice with beans) | 12 | 33.4 | 10.1 | 37.1 | 9.67 | 35.9 | 10.3 |
| Cooked rice with beans | 220 | 29.2 | 5.93 | 27.6 | 5.78 | 26.4 | 6.00 |
| Tofu (Soft tofu, Tofu stew, and Tofu in tofu stew) | 60 | 22.3 | 31.0 | 19.5 | 31.0 | 23.1 | 31.0 |
| Soy milk | 200 | 8.56 | 6.13 | 8.69 | 6.56 | 8.50 | 5.89 |
| Soybean paste soup/Fast-fermented bean paste/Soybean paste/Ssamjang | 9 | - | 16.2 | - | 16.0 | - | 16.3 |
| Cooked rice with multi-grains | 220 | - | 12.3 | - | 10.7 | - | 13.1 |
| Bean sprouts/Mung bean sprouts | 40 | - | 5.13 | - | 5.13 | - | 5.14 |
| **Proanthocyanidins** |  |  |  |  |  |  |  |
| Apple/Apple juice | 200 | 83.5 | 31.9 | 82.4 | 30.2 | 83.7 | 32.9 |
| Grapes/Grapes juice | 200 | 10.1 | 14.7 | 10.3 | 13.9 | 10.1 | 15.1 |
| Strawberries | 150 | - | 15.5 | - | 15.6 | - | 15.4 |
| Cooked rice with multi-grains | 220 | - | 12.5 | - | 12.4 | - | 12.6 |
| Peach/Plum | 100 | - | 5.18 | - | 5.07 | - | 5.24 |
| Green tea | 120 | - | - | - | 5.42 | - | - |

r squared values were determinant of variation and means the proportion of total variation in each antioxidant explained by the variation in intake from each food item. Intake (%) was the proportion of each antioxidant consumed from each food items out of the total intake of that antioxidant.
